# Dengue-Associated Mortality, Climate Anomalies, and Geographic Emergence in Peru, 2017–2024: A Spatiotemporal Study Triangulating Death Records and Epidemiological Surveillance

**DOI:** 10.3390/v18101051

**Published:** 2026-09-22

**Authors:** Victor J. Vera Ponce, Jhosmer Ballena-Caicedo, Julio David Sagastegui Jauregui, Nelly Del Carmen Villegas Ampuero, Fiorella E. Zuzunaga-Montoya

**Affiliations:** 1 Facultad de Medicina (FAMED), Universidad Nacional Toribio Rodriguez de Mendoza de Amazonas (UNTRM), Chachapoyas 01001, Amazonas, Peru; 7330178022@untrm.edu.pe (J.B.-C.); fiorella.zuzunaga@untrm.edu.pe (F.E.Z.-M.); 2 Escuela de Odontología, Universidad Nacional Toribio Rodriguez de Mendoza de Amazonas (UNTRM), Chachapoyas 01001, Amazonas, Peru; julio.sagastegui.epg@untrm.edu.pe (J.D.S.J.); nelly.villegas@untrm.edu.pe (N.D.C.V.A.)

**Keywords:** dengue, mortality, climate variability, death certificates, geographic emergence, spatiotemporal analysis, Peru

## Abstract

Dengue transmission has expanded across the Americas, but the spatiotemporal distribution of mortality recorded in death certificates and its ecological relationship with climate remain insufficiently characterized. **Objective**: To evaluate dengue-associated mortality recorded in Peruvian death certificates during 2017–2024, characterize geographic emergence across fixed provinces, estimate associations with local climate anomalies, and triangulate findings with epidemiological surveillance. **Methods**: We conducted a retrospective ecological spatiotemporal study combining the National Death Information System, national dengue surveillance, official population projections, gridded climate data, and a fixed 196-province framework. The primary outcome was an A90 or A91 code in any certificate position; province-month was the principal unit. Poisson models included a population offset, provincial fixed effects, calendar month, linear time, a 2020–2021 indicator, and temperature and precipitation anomalies at lags 0–3 months. **Results**: The updated snapshot contained 987 certificates with A90/A91 during 2017–2024; 968 were territorially eligible. Thirty-six provinces were classified as emergent. Surveillance contributed 772,387 notified cases, and department-year deaths and cases were correlated (Spearman ρ = 0.772). In the corrected primary model, the cumulative ecological mortality rate ratio comparing the 90th with the 50th percentile was 1.75 (95% CI: 1.41–2.17) for mean-temperature anomalies and 2.69 (95% CI: 1.97–3.67) for precipitation anomalies. Estimates remained positive under position-A, code-specific, syndromic, and temporal sensitivity definitions. **Conclusions**: Recorded dengue-associated mortality was concentrated during recent outbreaks and appeared in provinces without baseline-period mortality. Local climate anomalies preceded higher province-month mortality rates, but these ecological associations are compatible with indirect transmission and health-system pathways and do not establish direct individual-level effects.

## 1. Introduction

Dengue is a mosquito-borne arboviral disease and an increasing threat to global public health. Nearly half of the world’s population is at risk, and an estimated 100 to 400 million infections occur annually, with a clinical spectrum ranging from asymptomatic infection to severe dengue and death [1,2]. The reported burden has increased markedly over recent decades, and 2024 recorded the largest number of cases reported worldwide, with transmission in more than 100 countries and a predominant contribution from the Region of the Americas [1,3].

Dengue expansion is not driven by a single determinant. Transmission depends on competent vectors, particularly *Aedes aegypti* in urban and peri-urban settings, whose immature stages frequently develop in artificial containers and stored water in or around households. Inadequate water supply, water-storage practices, unplanned urbanization, human mobility, population density, and poverty-related infrastructure deficits therefore modify vector exposure and the probability of transmission and adverse clinical outcomes. Temperature can alter mosquito survival, biting activity, vector competence, and the extrinsic viral incubation period, whereas precipitation and humidity influence aquatic habitat availability and adult-vector persistence; these effects are nonlinear and context-dependent [1,4,5,6,7]. Any association with mortality is therefore expected to operate primarily through climate-sensitive transmission and downstream clinical and health-system pathways rather than as a direct effect of weather on individual deaths.

In Latin America and the Caribbean, dengue has recently reached historic levels. The Pan American Health Organization reported that 2024 was the largest regional epidemic since records began in 1980, with more than 12.6 million cases, more than 21,000 severe cases, and more than 7700 reported deaths [8]. Although early recognition and appropriate clinical management can keep dengue case fatality low, very large transmission waves can still produce substantial absolute mortality and strain health services [1,8,9]. In this context, incidence alone is insufficient to characterize dengue burden.

Peru is a relevant setting in which to study the interaction among transmission, climate, territory, and mortality. Dengue is endemic in the country, with seasonal transmission usually more intense between November and May; however, the 2023 outbreak showed an abrupt increase, affected historically active northern departments, and produced substantial increases in areas where local transmission had previously been limited, including Metropolitan Lima [10]. During the first 30 weeks of 2023, 222,620 cases and 381 dengue-related deaths were reported, with a substantial concentration of deaths among older adults; moreover, several high-burden territories were exposed to extreme rainfall linked to Cyclone Yaku [10]. Routine dengue surveillance in Peru is intended to detect and characterize notified transmission across time and territory, support outbreak alerts, and orient epidemiological response [11,12]. National technical standards separately define epidemiological and laboratory surveillance and entomological surveillance and vector-control activities [12,13], including surveillance of *Aedes aegypti* and the potential entry of *Aedes albopictus*. However, case-notification records reflect testing, reporting, and care-seeking processes and do not replace the evaluation of death certificates. Peru’s ecological heterogeneity, including Amazonian lowlands, northern coastal areas, arid coastal zones, and Andean highlands, makes a single national climate metric inappropriate. This heterogeneity motivated the use of province-month units, province-specific climate anomalies, and a fixed territorial framework rather than treating dengue-associated mortality as a spatially uniform process.

Available evidence from Peru has described dengue outbreaks, incidence, clinical characteristics, and epidemiological surveillance; however, important gaps remain regarding mortality recorded in death certificates, its emergence across fixed provinces, its temporal relationship with local climate anomalies, and its concordance with surveillance. Mortality records, surveillance notifications, climate series, and territorial identifiers capture different components of the same epidemic process. Integrating these sources makes it possible to evaluate whether recorded mortality mirrors notified transmission, whether provinces without baseline-period mortality record events during recent outbreaks, and whether local climate anomalies precede increases in dengue-associated mortality. Therefore, this study aimed to evaluate spatiotemporal patterns of dengue-associated mortality recorded in Peru during 2017–2024, estimate its ecological association with local climate anomalies, characterize geographic emergence across a fixed province framework, and triangulate findings with epidemiological surveillance data.

## 2. Materials and Methods

### 2.1. Study Design

We conducted a retrospective ecological spatiotemporal observational study using secondary mortality, surveillance, climate, population, and territorial data to evaluate dengue-associated mortality recorded in death certificates in Peru between 1 January 2017 and 31 December 2024. The corrected analysis used one updated SINADEF snapshot for the entire study period rather than appending later months to the earlier extract. All 96 calendar months were represented. However, the source copy did not preserve an official download date or certificate-level registration and update timestamps; consequently, calendar coverage through December 2024 can be documented, but the administrative exhaustiveness or definitive closure of the 2024 microdata cannot be certified. Manuscript preparation followed the STROBE recommendations for observational studies [14], and the complete checklist is presented in Table S1.

### 2.2. Data Sources

The primary source was the National Death Information System (Sistema Informático Nacional de Defunciones; SINADEF), which contains death certificates and causes recorded in six positions and coded according to the International Classification of Diseases, Tenth Revision (ICD-10) [15]. For epidemiological triangulation, we used the public dengue surveillance database of the National Center for Epidemiology, Prevention and Disease Control of the Ministry of Health of Peru (CDC/MINSA), which reports notified and severe cases with temporal and territorial information [11].

Population denominators were obtained from projections of the National Institute of Statistics and Informatics (INEI) at provincial level for 2018–2024; the 2018 population was carried back to 2017 as an operational approximation [16]. Climate exposures were derived from the National Aeronautics and Space Administration Prediction of Worldwide Energy Resources (NASA POWER) monthly data for provincial centroids, using 1991–2020 as the reference climatology [17]. The Database of Global Administrative Areas (GADM), version 4.1, was used only as auxiliary geometry, whereas the official INEI territorial catalog and UBIGEO codes defined the canonical province framework [18].

### 2.3. Population and Analytical Unit

The source population comprised all deaths dated within the study period in the updated SINADEF snapshot. For geographic analyses, we retained certificates with a valid date, residence in Peru, and identifiable department, province, and district. All sources were harmonized to a single fixed framework of 196 INEI provinces using UBIGEO codes, normalized exact matches, and explicitly audited aliases. Foreign records and certificates with unrecorded or unidentifiable critical geography were excluded from geographic analyses and retained in the record-level lineage. A complete 196-province by 96-month skeleton was constructed before population, climate, surveillance, and mortality data were joined by left joins. Geographic emergence therefore denotes the appearance of recorded deaths across fixed analytical provinces; it does not refer to changes in administrative boundaries. The primary analytical unit was province-month. Figure S2 presents the conceptual directed acyclic graph used to guide interpretation without implying individual-level causal effects.

### 2.4. Outcome

The primary outcome was dengue-associated death recorded in SINADEF, defined by ICD-10 code A90 or A91 in any of the certificate positions A–F. A90 corresponds to dengue fever and A91 to dengue hemorrhagic fever. This multiple-cause definition was selected to capture certificates in which dengue appeared anywhere in the recorded causal sequence or as a contributing condition; it was not intended to identify confirmed dengue-attributable deaths. Position A was evaluated as a more restrictive positional sensitivity outcome. Because the first recorded position may represent the immediate cause and the underlying cause requires formal mortality-coding selection rules, position A was not treated as synonymous with the underlying cause.

Additional outcome definitions were any A90, any A91, A90 only, A91 only, and simultaneous A90/A91 mention. Two clinically compatible sensitivity outcomes required an A90/A91 mention plus at least one ICD-10 prefix from frozen clinical groups: a broad definition including shock, hemorrhage or coagulopathy, sepsis, renal failure, hepatic failure, or respiratory failure, and a narrow definition restricted to shock or hemorrhage/coagulopathy. Exact codes are provided in Table S4. Text matching was retained only as an exploratory analysis and excluded the text line paired with an A90/A91 code, preventing the term “dengue hemorrhagic fever” from being recycled as independent hemorrhagic evidence. No dated protocol or commit predating outcome inspection was available to demonstrate prespecification of the syndromic definitions; they were therefore interpreted as sensitivity analyses rather than confirmatory validation.

Analytical endpoints were aligned with the study objectives. Spatiotemporal patterns were evaluated using monthly and annual counts, rates, certificate characteristics, and geographic distributions. Geographic emergence was assessed with fixed-province categories comparing the 2017–2019 baseline with events recorded in 2023–2024 and requiring notified transmission during the recent period for the emergent category. Climate-mortality associations were evaluated as cumulative mortality rate ratios comparing the 90th with the 50th percentile of local climate anomalies across lags 0–3 months. Surveillance triangulation used aggregated count comparisons, Spearman correlations, and peak-month differences. Alternative ICD-10 and temporal definitions were treated as sensitivity analyses. Table 1 summarizes the study objectives, operational endpoints, and corre-sponding analyses.

### 2.5. Climate Exposures

The primary exposures were monthly mean-temperature anomaly and monthly cumulative-precipitation anomaly. Temperature anomalies were calculated as observed temperature minus the 1991–2020 mean for the same province and calendar month. Precipitation was transformed with log(1 + monthly precipitation) before subtraction of the corresponding province-calendar-month climatological mean. Both anomalies were standardized using their distributions in the corrected 2017–2024 province-month panel. The 90th-versus-50th-percentile contrasts were fixed from the main lag-eligible panel and held constant across outcome sensitivities. Humidity and maximum temperature were retained for descriptive correlation assessment because of expected collinearity among meteorological variables.

### 2.6. Model Covariates and Estimation

Models included the logarithm of annual provincial population as an offset. Seasonality was represented by calendar-month indicators, secular change by a linear time index beginning in January 2017, and the pandemic period by an indicator for 2020–2021. Provincial fixed effects controlled for all time-invariant territorial characteristics. Temperature and precipitation were entered simultaneously with separate unconstrained coefficients at lags 0, 1, 2, and 3 months.

The 2020–2021 indicator was intended only to absorb a broad period-level shift and was not assumed to capture every pandemic-related change in health-care use, surveillance, diagnosis, hospital capacity, or death certification. Robustness was therefore evaluated by refitting the main model after excluding 2020–2021 while retaining the original calendar-time index.

### 2.7. Triangulation with Epidemiological Surveillance

CDC/MINSA records were harmonized to the same province-month framework and aggregated additionally at department-year level. The canonical surveillance series contained 772,387 notified cases and 2775 severe cases during 2017–2024, with no resolvable territorial units left unmatched. This comparison was framed as aggregated triangulation rather than validation because no nominal linkage was available between death certificates and case notifications. Metrics included Spearman correlation, differences between annual peak months, and descriptive ratios of recorded deaths to notified cases.

### 2.8. Statistical Analysis

First, certificates with A90/A91 were described by age, sex, place of death, ICD-10 composition and first position, coding depth, autopsy status, and syndromic co-mentions. Blank later positions were described as unpopulated rather than automatically classified as missing because a valid certificate need not contain six conditions. Home and health-facility deaths were compared for the probability that A90/A91 first appeared in positions B–F using modified Poisson models with robust variance; the adjusted model included year, age group, and sex and standardized marginal predictions were used for the adjusted absolute difference.

Second, annual national rates were calculated as the sum of territorially eligible deaths divided by the summed provincial population. Provinces with at least one death during 2017–2019 were classified as historically active. Provinces with no baseline deaths but at least one death and notified transmission during 2023–2024 were classified as emergent. Remaining provinces with at least one death were classified as recurrent or intermittent, and provinces without deaths were classified as having no recorded mortality. Sensitivities required at least two recent deaths or events in at least two recent months and restricted classification to 2017–2023.

Third, associations between climate anomalies and dengue-associated mortality counts were estimated using Poisson models at the province-month level. The main model used the population offset, province fixed effects, calendar month, linear time, the pandemic indicator, and temperature and precipitation at lags 0–3 months. Confidence intervals used robust sandwich standard errors clustered by province. The lag-eligible candidate panel contained 18,228 province-months and 959 deaths; provinces without within-province outcome variation did not contribute to fixed-effect estimation and were counted explicitly.

Because the models used counts with a population offset, exponentiated contrasts were interpreted as province-level mortality rate ratios (MRRs). They are ecological rate ratios and must not be interpreted as individual-level relative risks. Cumulative MRRs compared the 90th with the 50th percentile of each anomaly and incorporated the covariance among all four lag coefficients.

Notified cases were not included as a standard covariate in the primary climate model because they are downstream of climate-sensitive transmission and could mediate part of the pathway from climate to severe disease and death. Death-to-case ratios were therefore reported only as aggregated descriptive measures and not as individually linked case-fatality estimates.

Fourth, a closed sensitivity family repeated the identical model specification for position A, any A90, any A91, A90 only, A91 only, broad and narrow ICD-only syndromic definitions, exclusion of 2020–2021, and complete calendar years 2017–2023. Component-removal analyses reconstructed the broad syndromic outcome as the union of the remaining clinical groups. Model convergence, effective provinces and cells, observed and predicted events, Pearson statistic per degree of freedom, and territorial concentration were documented. Analyses were performed in Python 3.12.13 using pandas 2.3.3, NumPy 2.3.5, SciPy 1.16.3, and statsmodels 0.14.6; figures were generated using Matplotlib 3.10.8. Reproducible scripts, quality-assurance records, and aggregated analytical outputs are provided as supplementary files.

**Table 1:** Study objectives, operational endpoints, and analyses.

Study Objective	Operational Endpoint/Estimand	Unit	Primary Analysis	Role
Spatiotemporal mortality patterns	Monthly and annual counts, population rates, and demographic/certification profile	Province-month and individual certificates	Descriptive analysis with INEI population denominators	Primary
Geographic emergence	Fixed-province category: historically active, recurrent/intermittent, emergent, or no recorded mortality	Province	2017–2019 baseline versus 2023–2024; robust and 2017–2023 sensitivities	Primary
Surveillance triangulation	Aggregated concordance, peak-month difference, and descriptive death-to-case ratios	Department-year and province-month	Aggregated comparison without nominal linkage	Primary triangulation
Climate-mortality association	Cumulative MRR for q90 versus q50 of anomalies across lags 0–3 months	Province-month	Poisson fixed-effects distributed-lag model	Primary inferential analysis
Outcome and temporal robustness	Position A, A90/A91 code-specific and ICD-only syndromic outcomes; exclusion of 2020–2021; 2017–2023	Certificates and province-month	Closed sensitivity model family	Sensitivity analysis

Note: CDC/MINSA = National Center for Epidemiology, Prevention and Disease Control/Ministry of Health of Peru; ICD-10 = International Classification of Diseases, Tenth Revision; INEI = National Institute of Statistics and Informatics; MRR = mortality rate ratio; SINADEF = National Death Information System.

### 2.9. Ethical Considerations

The study used exclusively secondary, anonymized or aggregated data from administrative systems and public repositories. No primary information was collected, no individuals were contacted, and no individual identifiers were accessed. Because this was a secondary analysis of non-identifiable data, the study was not submitted for institutional ethics committee review. The analysis was conducted in accordance with principles applicable to research using secondary data, including confidentiality protection and proportional use of available information [19].

## 3. Results

### 3.1. Sample Selection and Construction of the Analytical Panel

The updated SINADEF snapshot contained 1,285,955 death records dated from 1 January 2017, through 31 December 2024. We identified 987 certificates with A90 or A91 in at least one of positions A–F. Nineteen were excluded from geographic analyses under mutually exclusive primary reasons: 17 had unpopulated or unidentifiable critical geography and two had a foreign or unrecorded country of residence. The remaining 968 certificates were represented in the balanced 196-province by 96-month panel. Population was available for all 18,816 cells. Complete climate lags 0–3 were available for 18,228 cells containing 959 deaths; the primary fixed-effects model retained 92 provinces with outcome variation and 8556 cells while preserving all 959 lag-eligible deaths. The corrected flow is presented in Figure S1.

All external sources were linked to the fixed INEI province framework by audited left joins. The corrected surveillance series contributed 772,387 notified cases and 2775 severe cases; no resolvable surveillance units remained unmatched. Population and climate covered all 196 provinces and 96 months before the first three months per province were lost by construction of lags 0–3. Data sources and provenance are summarized in Table S2, the analytical dictionary in Table S3, and the complete flow and unit counts in Table S7.

### 3.2. Record Quality and Analytical Unit

Among the 987 A90/A91 certificates, the median number of populated ICD-10 positions was 3.0 (IQR: 2.0–4.0). Across years, 14.1% to 25.9% contained A90/A91 in position A, whereas 74.1% to 87.9% first mentioned A90/A91 in positions B–F. Position-population percentages for all SINADEF deaths and coding-depth summaries for A90/A91 certificates are reported separately; unpopulated positions B–F were not treated as conventional missing values. Annual results are shown in Figure S3 and Table S5.

The corrected complete panel contained 18,816 province-month cells; after construction of lags 0–3, 18,228 candidate cells remained. Of these, 94.0% had no recorded dengue-associated death. The fixed-effects estimator retained only provinces with within-province outcome variation, a mechanical requirement documented separately from record-level exclusions. This distinction prevents the former reduction from 849 to 529 deaths from being misrepresented as sequential certificate exclusion.

### 3.3. Clinical–Demographic and Certification Characteristics

Among the 987 certificates, median age was 58.0 years (IQR: 32.0–76.0), with age unavailable in six. Men accounted for 532 certificates (53.9%), women for 454 (46.0%), and sex was unrecorded in one. Adults aged 75 years or older represented 270 certificates (27.4%), followed by those aged 60–74 years with 207 (21.0%). The updated profile is presented in Table 2.

The most frequent place of death was a health facility, with 802 certificates (81.3%), followed by home, with 143 (14.5%). Among home deaths, A90/A91 first appeared in positions B–F in 95 of 143 certificates (66.4%), compared with 680 of 802 facility deaths (84.8%). The unadjusted prevalence ratio for a later first position was 0.784 (95% CI: 0.695–0.884); after adjustment for year, age group, and sex, it was 0.779 (95% CI: 0.692–0.877), with an adjusted standardized absolute difference of −18.8 percentage points. Position A was correspondingly more frequent at home (33.6%) than in facilities (15.2%). Laboratory confirmation, previous clinical evaluation or hospitalization, certifying-physician information, and linkage to clinical records were unavailable. Seven certificates recorded that an autopsy had been performed, all in health facilities. Detailed comparisons are provided in Table S12b and Figure S12.

A90 appeared in any position in 384 certificates (38.9%) and A91 in 609 (61.7%); 378 were A90 only, 603 A91 only, and six contained both codes. A90/A91 appeared in position A in 178 certificates (18.0%). The first mention occurred in position C in 323 (32.7%), B in 194 (19.7%), D in 193 (19.6%), A in 178 (18.0%), E in 82 (8.3%), and F in 17 (1.7%). The broad ICD-only syndromic definition identified 761 certificates (77.1%) and the narrow shock/hemorrhage definition 409 (41.4%); exploratory text matching added 15 certificates and was not used in primary syndromic models. Definitions and model-based sensitivities are reported in Tables S4 and S12 and Figure S6.

**Table 2:** Clinical–demographic and certification profile of dengue-associated death certificates, 2017–2024.

Indicator	Result
Certificates with A90/A91 in any position	987
Age, median (IQR), years	58.0 (32.0–76.0)
Age unrecorded	6 (0.6)
Sex: Male	532 (53.9)
Sex: Female	454 (46.0)
Sex: Unrecorded	1 (0.1)
Age group: ≥75 years	270 (27.4)
Age group: 60–74 years	207 (21.0)
Age group: 45–59 years	150 (15.2)
Age group: 30–44 years	135 (13.7)
Age group: 15–29 years	124 (12.6)
Age group: 5–14 years	56 (5.7)
Age group: <5 years	39 (4.0)
Place of death: Health facility	802 (81.3)
Place of death: Home	143 (14.5)
Place of death: Other	21 (2.1)
Place of death: In transit	17 (1.7)
Place of death: No record	3 (0.3)
Place of death: Public road	1 (0.1)
A90 in any position	384 (38.9)
A91 in any position	609 (61.7)
A90 only	378 (38.3)
A91 only	603 (61.1)
Both A90 and A91	6 (0.6)
A90/A91 in position A	178 (18.0)
Number of populated ICD-10 positions, median (IQR)	3.0 (2.0–4.0)
First A90/A91 position: A	178 (18.0)
First A90/A91 position: B	194 (19.7)
First A90/A91 position: C	323 (32.7)
First A90/A91 position: D	193 (19.6)
First A90/A91 position: E	82 (8.3)
First A90/A91 position: F	17 (1.7)
Broad ICD-only syndromic definition	761 (77.1)
Narrow ICD-only syndromic definition	409 (41.4)
Exploratory text-augmented definition	776 (78.6)
Autopsy recorded: Yes	7 (0.7)
Autopsy recorded: No	925 (93.7)
Autopsy: No record	55 (5.6)

Note: Values are *n* (%) unless otherwise stated. Percentages use 987 certificates as the denominator and may not sum to 100% because of rounding or unrecorded values. A90 = dengue fever; A91 = dengue hemorrhagic fever; ICD-10 = International Classification of Diseases, Tenth Revision; IQR = interquartile range.

### 3.4. Temporal Evolution and Geographic Emergence

The annual number of certificates with A90/A91 in any position was 64 in 2017, 16 in 2018, 27 in 2019, 131 in 2020, 116 in 2021, 134 in 2022, 376 in 2023, and 123 in 2024. The highest monthly counts were 108 in June 2023 and 97 in May 2023; March 2024 contributed 56 certificates. Among the 968 territorially eligible certificates used for population rates, annual counts were 60, 16, 27, 130, 116, 134, 363, and 122, respectively. The corresponding national rate was highest in 2023 at 1.08 per 100,000 population. Temporal patterns are shown in Figure 1, annual rates in Table S6, department-year estimates in Table S6b, and the complete 2024 series in Figure S11 and Table S19.

The largest territorially eligible counts occurred in Piura (222), Lambayeque (113), Madre de Dios (103), Ica (99), San Martín (58), Loreto (58), Junín (52), Lima (48), La Libertad (47), Ucayali (41), and Tumbes (35). At the province level, the highest counts were recorded in Piura, Piura (130); Tambopata, Madre de Dios (95); Chiclayo, Lambayeque (75); Chincha, Ica (59); Lima Metropolitana, Lima (38); and Coronel Portillo, Ucayali (36).

The fixed-province classification identified 29 historically active provinces, 27 recurrent or intermittent provinces, 36 emergent provinces, and 104 provinces with no recorded mortality. Under the more restrictive requirement of at least two recent deaths or events in at least two recent months, 19 provinces remained emergent; the 2017–2023 sensitivity analysis identified 23 emergent provinces. The spatial distribution is shown in Figure 1, and category sensitivities in Figure S10.

### 3.5. Triangulation with Epidemiological Surveillance

The canonical CDC/MINSA surveillance series contributed 772,387 notified dengue cases and 2775 severe cases during 2017–2024. The balanced panel contained 968 territorially eligible dengue-associated deaths. At the department-year level, recorded deaths and notified cases were correlated (Spearman ρ = 0.772; *p* < 1 × 10^−40^). Figure 2 presents the national monthly series on separate axes and the department-year relationship; summary metrics and the complete department-year data are provided in Table S9a,b.

Among 102 department-years with both deaths and notified cases, the difference between the death peak and the case peak had a median of 0 months (IQR: −2 to 1). Lag-specific national correlations are presented in Figure S7. Descriptive ratios of recorded deaths per 1000 notified cases are reported in Table S17 and must not be interpreted as individually linked case-fatality estimates.

### 3.6. Association Between Climate Anomalies and Dengue-Associated Mortality

Climate anomalies varied across provinces and months. The primary model used 18,228 lag-eligible candidate cells containing 959 deaths; after provinces without outcome variation were removed by fixed-effect estimation, 8556 cells from 92 provinces remained and still contained all 959 deaths. Mean-temperature and precipitation anomalies were entered simultaneously. Their distributions and correlations are presented in Table S8 and Figure S5.

In the corrected primary model, the 90th-versus-50th-percentile contrast in mean-temperature anomaly was associated with a cumulative ecological MRR of 1.75 across lags 0–3 months (95% CI: 1.41–2.17). The corresponding precipitation contrast was associated with an MRR of 2.69 (95% CI: 1.97–3.67). Lag-specific estimates and cumulative contrasts are presented in Figure 3 and Table S11.

Lag-specific temperature estimates were largest at lags 0 and 1, whereas precipitation estimates were positive across lags 0–3. These estimates represent within-province ecological associations between local deviations from expected climate and recorded mortality rates. They do not indicate that weather directly caused individual deaths.

### 3.7. Diagnostics and Sensitivity Analyses

The primary corrected model predicted 959.0 deaths for 959 observed deaths and converged with finite parameters and covariance estimates. The Pearson statistic per degree of freedom was 1.48, and the province with the largest event count contributed 13.1% of events. Model-specific diagnostics, effective cells, provinces, event counts, and concentration measures are reported in Table S15.

Results were directionally consistent across the closed sensitivity family. For A90/A91 in position A, cumulative MRRs were 1.72 (95% CI: 1.18–2.49) for temperature and 3.29 (1.84–5.88) for precipitation. Estimates were 2.04 (1.61–2.58) and 3.03 (2.37–3.88) for any A90, and 1.61 (1.25–2.07) and 2.44 (1.64–3.60) for any A91. Broad and narrow ICD-only syndromic definitions yielded temperature MRRs of 1.64 and 1.69 and precipitation MRRs of 2.52 and 2.16, respectively. After excluding 2020–2021, the MRRs were 1.61 (1.26–2.07) and 2.29 (1.57–3.34); restricting the analysis to complete years 2017–2023 yielded 2.01 (1.53–2.64) and 2.34 (1.74–3.15). Full estimates, including A90-only, A91-only, and component-removal analyses, are presented in Tables S12–S14 and Figure 4.

## 4. Discussion

### 4.1. Main Findings

In this national ecological study, the updated SINADEF snapshot contained 987 dengue-associated death certificates during 2017–2024, with marked concentration in 2023 and continued events across the full 2024 calendar year. On a corrected fixed framework of 196 provinces, 36 provinces met the main definition of geographic emergence. Mortality showed aggregated concordance with notified cases, and within-province temperature and precipitation anomalies preceded higher recorded mortality rates across lags of 0–3 months. The principal estimates remained positive under position-A, A90/A91-specific, ICD-only syndromic, pandemic-exclusion, and complete-year sensitivities.

### 4.2. Comparison with Other Studies

The findings are consistent with the increasing and geographically redistributing global dengue burden. Global burden estimates show that dengue deaths vary substantially across regions, ages, and information systems [20], while global suitability maps describe a dynamic spatial frontier influenced by urbanization, mobility, and climate [21]. The present analysis adds a mortality-record perspective and, by using a fixed INEI province framework, distinguishes geographic emergence of recorded mortality from changes in administrative boundaries.

The short-lag climate associations are compatible with established transmission mechanisms rather than a direct lethal action of weather. Temperature modifies vector competence, mosquito survival, and the extrinsic incubation period; precipitation and humidity influence breeding habitat and vector persistence [5,6,7]. Models and projections have also shown climate-sensitive redistribution of *Aedes*-borne transmission risk [6,22]. In this study, climate could affect mortality indirectly through transmission intensity, the number of severe cases reaching services, clinical pressure, and subsequent certification. The province-month MRRs therefore cannot be translated into individual risks or interpreted as proof that a particular anomaly caused an individual death.

Peruvian evidence has linked temperature and ENSO-related conditions with dengue outbreak risk and has documented strong provincial heterogeneity during previous epidemics [7,23]. The corrected results likewise showed temporal concentration and emergence across fixed provinces, including locations without baseline-period recorded mortality. Nevertheless, the emergent classification is a surveillance-informed descriptive construct: it indicates new recorded mortality under the prespecified temporal rule, not proof of new biological establishment, prior absence of transmission, or a change in provincial borders.

Certification analyses add an important qualification. Home deaths were less likely than facility deaths to have A90/A91 first recorded in later positions and more likely to contain the code in position A, but these differences do not establish greater diagnostic certainty because laboratory confirmation, previous clinical evaluation, hospitalization, certifier information, and medical-record linkage were unavailable. Similarly, positive results under broad and narrow ICD-only syndromic definitions support robustness to compatible co-mentions but cannot establish dengue attribution because shock, sepsis, organ failure, and hemorrhagic conditions are not etiologically specific. Prior reviews likewise emphasize that fatal outcomes reflect interactions among clinical recognition, referral, health-system conditions, and transmission burden [9,24].

### 4.3. Public Health and International Implications

First, the results illustrate how death-certificate mortality, case surveillance, and local climate data can be evaluated jointly without conflating their meanings. Surveillance captures notified transmission; SINADEF captures recorded mortality and certification practices; climate variables represent temporally ordered ecological exposures. Their triangulation can identify periods and provinces warranting closer assessment, but this study did not evaluate the effectiveness of surveillance, vector-control, clinical, or hospital-capacity interventions. Comparative studies likewise show that passive surveillance can underestimate dengue burden to differing degrees across Latin American settings [25].

Second, the 36 provinces meeting the main emergent definition represent settings with recent recorded mortality and notified transmission after no baseline-period deaths. They warrant review alongside routine epidemiological and entomological surveillance and local clinical information. The analysis does not demonstrate delayed diagnosis, weaker referral systems, lower prior experience, or facility saturation in those provinces because those mechanisms were not measured.

Third, the fully auditable 196-province panel and the explicit separation between certificate exclusions, lag construction, and fixed-effect estimation address a common reproducibility problem in multisource ecological analyses. The framework is portable to other settings with unlinked mortality, surveillance, and climate systems, provided that geographic crosswalks, denominators, temporal coverage, and the interpretation of multiple-cause mortality are documented rather than treated as clerical details.

### 4.4. Limitations

This study has limitations. First, its retrospective ecological design does not identify individual risks or causal effects. Although climate exposures preceded the recorded deaths, residual and time-varying confounding remains possible, including vector-control activity, circulating serotypes, primary versus secondary infection, immunity, mobility, health-care capacity, diagnostic practice, and outbreak-related changes in surveillance or certification. The binary 2020–2021 indicator cannot represent all pandemic mechanisms; the exclusion analysis reduced this concern but did not remove other temporal biases. Second, the primary multiple-cause outcome increases sensitivity but may include certificates in which dengue was a contributing condition rather than the underlying cause. Position A is not equivalent to the formally selected underlying cause, and later cause fields being unpopulated are not necessarily missing data. Coding density and place of death may affect the opportunity to identify an A90/A91 mention.

Third, SINADEF did not provide laboratory confirmation, prior health-care use or hospitalization, certifier information, or linkage to medical records; diagnostic certainty and care pathways could not therefore be assessed, especially for home deaths. The broad and narrow syndromic outcomes were ICD-only sensitivity definitions and remained etiologically nonspecific; no dated evidence was available to demonstrate that they were prespecified before outcome inspection. Fourth, mortality and surveillance data were not nominally linked, so their correlation and death-to-case ratios assess only aggregated concordance. Fifth, provincial-centroid climate values cannot capture urban microclimates or intraprovincial variation, and 2018 population was carried back to 2017. Finally, the updated snapshot contains deaths across all 12 months of 2024, but its exact official download date and certificate-level registration or update timestamps were unavailable; administrative exhaustiveness and definitive closure of 2024 cannot be certified.

## 5. Conclusions

By integrating death certificates, epidemiological surveillance, local climate anomalies, and a fixed official territorial framework, this study identified a substantial concentration of recorded dengue-associated mortality during recent outbreaks and geographic emergence in provinces without baseline-period mortality. Department-year mortality was concordant with notified transmission, and higher local temperature and precipitation anomalies were associated with higher province-month mortality rates over subsequent months.

These results should be interpreted as ecological associations with recorded dengue-associated mortality, not as direct weather effects on individual deaths or proof of dengue attribution for every certificate. The consistent position-A, code-specific, syndromic, and temporal sensitivities support analytical robustness, while the certification and provenance limitations define the boundaries of inference. The findings provide complementary evidence for local epidemiological assessment and generate testable hypotheses for linked clinical, surveillance, and mortality studies.

## Figures and Tables

**Figure 1:**
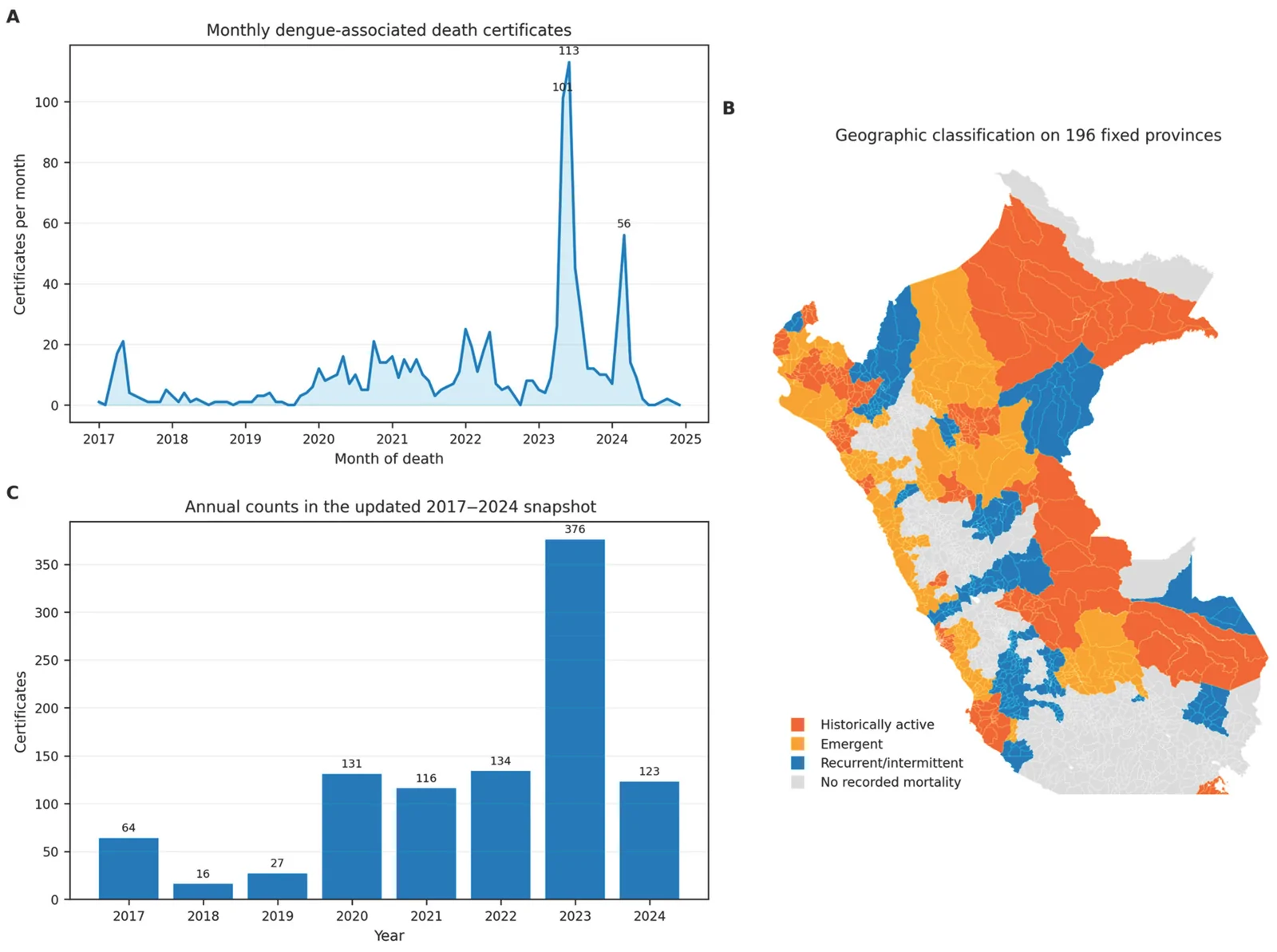
Temporal and geographic patterns of dengue-associated mortality, 2017–2024. (**A**) Monthly counts among all 987 certificates with A90/A91. (**B**) Main geographic classification on the fixed framework of 196 INEI provinces. (**C**) Annual certificate counts in the updated snapshot. Geographic emergence required no deaths during 2017–2019, at least one death during 2023–2024, and recent notified transmission.

**Figure 2:**
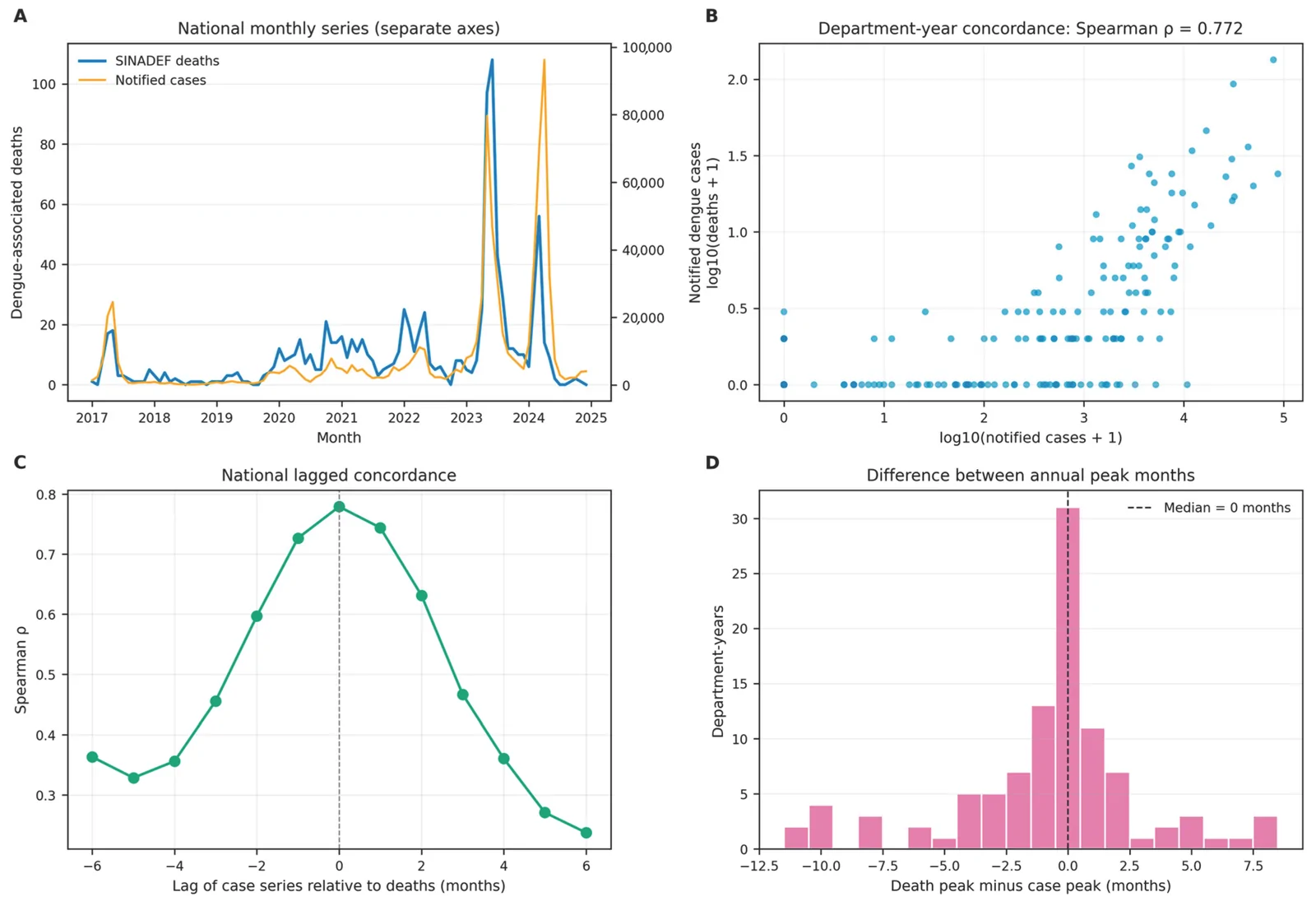
Triangulation of SINADEF dengue-associated mortality with CDC/MINSA epidemiological surveillance. (**A**) National monthly deaths and notified cases are shown using separate labeled y-axes; neither series is rescaled. (**B**) Department-year concordance; each blue point represents one department-year, with axes displaying log10(notified cases + 1) and log10(deaths + 1). Darker points result from overlapping semitransparent markers and do not denote a separate group. (**C**) Lagged national Spearman correlations; the vertical dashed line marks zero lag, corresponding to cases and deaths in the same calendar month. (**D**) Difference between annual death and case peak months. The dashed line marks the median difference of 0 months. CDC/MINSA = National Center for Epidemiology, Prevention and Disease Control/Ministry of Health of Peru; SINADEF = National Death Information System.

**Figure 3:**
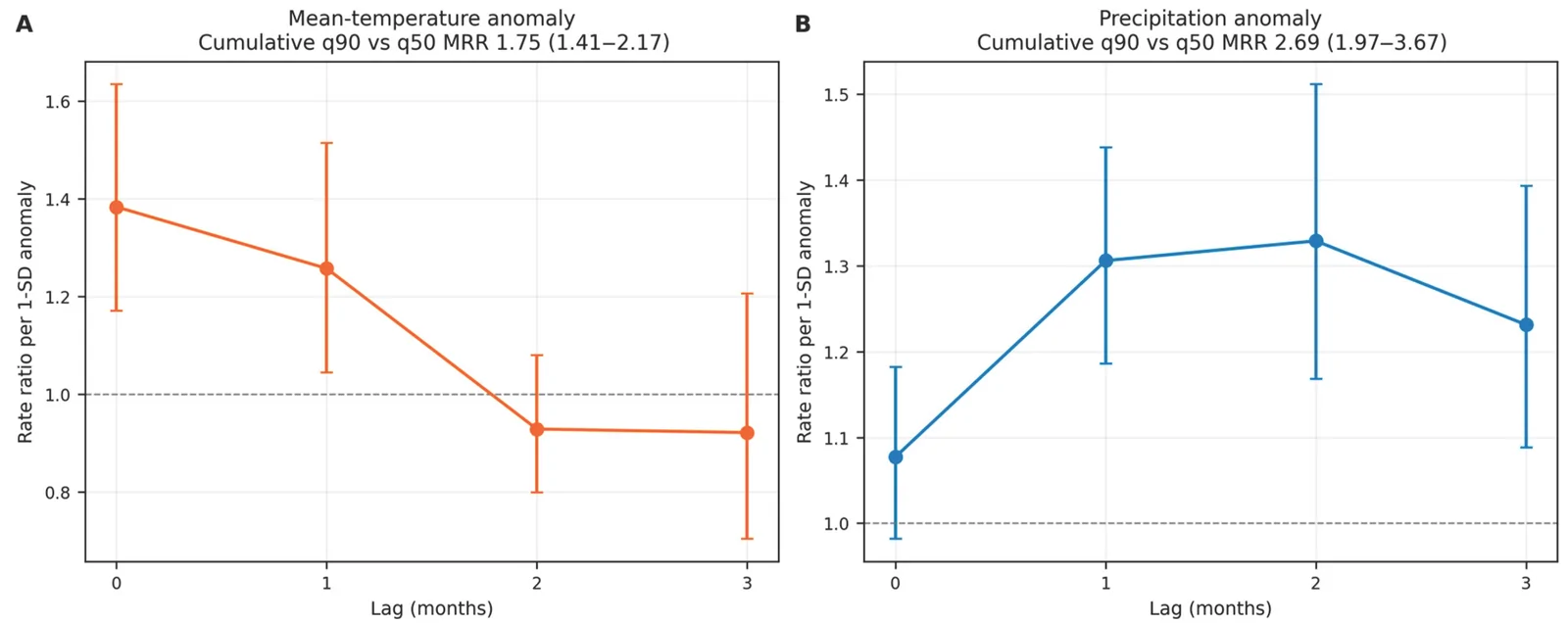
Lag-specific associations between climate anomalies and dengue-associated mortality. (**A**) Mean-temperature anomaly. (**B**) Precipitation anomaly. Points show province-month rate ratios per one-standard-deviation anomaly at each lag; subtitles report cumulative mortality rate ratios (MRRs) comparing the 90th with the 50th percentile across lags 0–3. Models include temperature and precipitation simultaneously, a population offset, provincial fixed effects, calendar month, linear time, and a 2020–2021 indicator, with standard errors clustered by province. Vertical error bars indicate 95% confidence intervals, and the horizontal dashed lines mark a rate ratio of 1, representing the null association.

**Figure 4:**
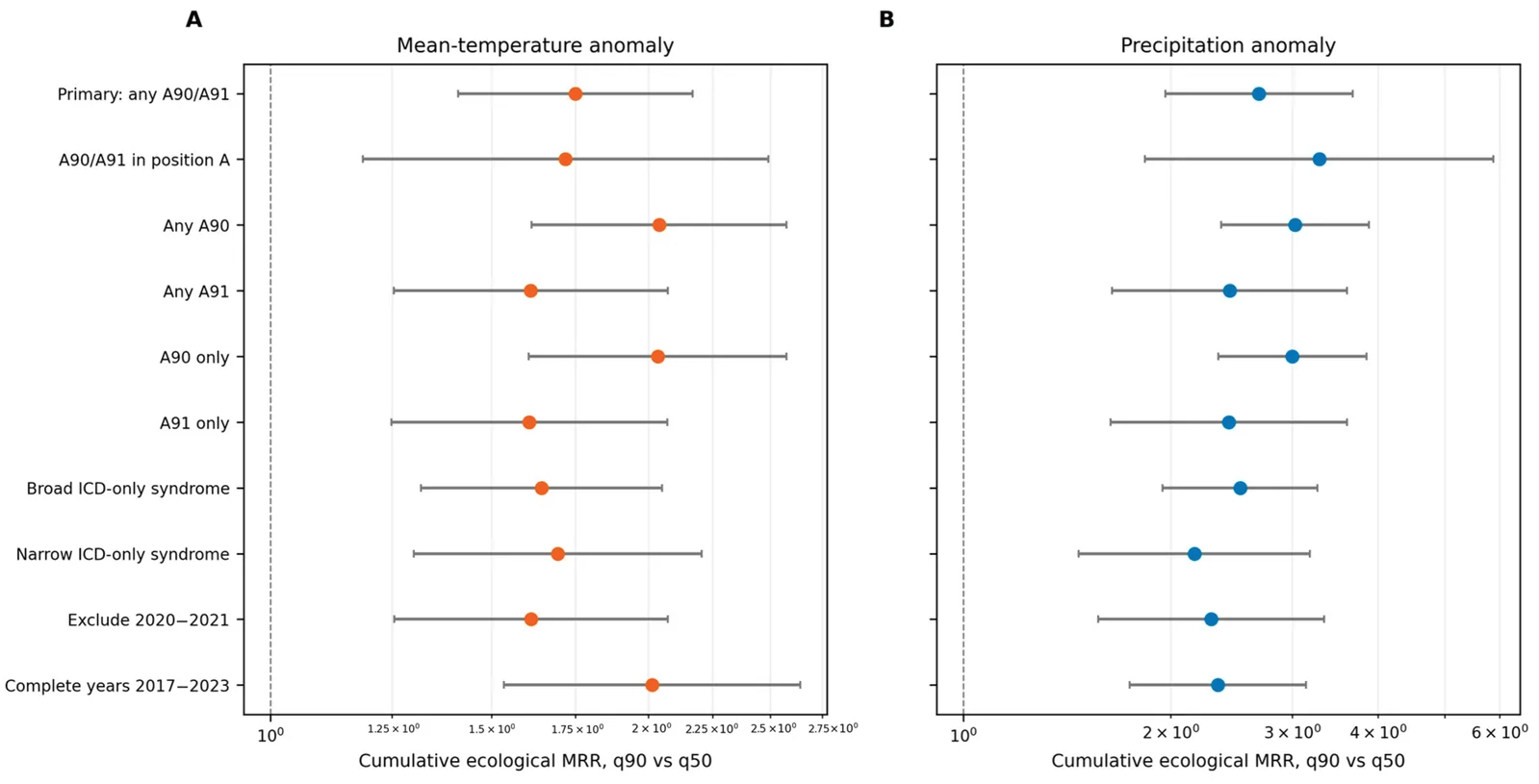
Sensitivity analyses for cumulative ecological mortality rate ratios comparing the 90th with the 50th percentile of local climate anomalies across lags 0–3. (**A**) Mean-temperature anomaly. (**B**) Precipitation anomaly. Points indicate cumulative ecological mortality rate ratios, horizontal error bars indicate 95% confidence intervals, and vertical dashed lines mark a mortality rate ratio of 1. The primary outcome was A90/A91 in any position. All outcome sensitivities used the same panel, exposure definitions, contrasts, offset, temporal terms, and fixed-effect structure. The temporal sensitivities excluded 2020–2021 or restricted follow-up to 2017–2023.

## Data Availability

The public surveillance, population, NASA POWER, and GADM sources are identified in the References. Aggregated analytical panels, model outputs, tables, figures, scripts, input manifests, QA records, and reproducibility logs are provided in the supplementary reproducibility archive. Certificate-level SINADEF records are not redistributed in order to respect restrictions applicable to the source microdata; access to the source must follow the procedures of the data provider.

## Supplementary Materials

The following supporting information can be downloaded at: https://www.mdpi.com/article/10.3390/v18101051/s1, Table S1. STROBE checklist; Table S2. Data sources, role, temporal coverage, and provenance limitations; Table S3. Analytical dictionary and recoding; Table S4. Outcome definitions and exact ICD-10 syndromic code groups; Table S5. ICD-10 position population and coding depth by year; Table S6a. Annual territorially eligible deaths, population, and mortality rates; Table S6b. Department-year dengue-associated mortality and population rates; Table S7. Corrected analytical flow and canonical unit counts; Table S8. Climate-exposure definitions, contrasts, and descriptive statistics; Table S9a. Summary of aggregated SINADEF–surveillance concordance; Table S9b. Department-year mortality and epidemiological-surveillance counts; Table S10. Complete computational specification of the climate model; Table S11. Corrected primary climate-model estimates; Table S12a. Climate-model sensitivity analyses by outcome definition; Table S12b. Home versus health-facility certification characteristics and modified-Poisson estimates; Table S13. Climate-model sensitivity analyses by temporal window; Table S14. Leave-one-component-out syndromic sensitivity analyses and unit tests; Table S15. Model diagnostics and effective analytical samples; Table S16. Information availability for home deaths and manual coding audit; Table S17. Descriptive recorded deaths per 1000 notified cases by department, 2017–2024; Table S18. Reproducibility and quality-assurance summary; Table S19. Full-year 2024 reconciliation across analytical populations and surveillance; Figure S1. Corrected sequential study flow for the updated analysis; Figure S2. Conceptual directed acyclic graph for interpretation of climate, transmission, care, death, certification, and surveillance; Figure S3. ICD-10 cause-position population and coding depth by year; Figure S4. Construction of the corrected province-month analytical panel; Figure S5. Correlations among standardized climate anomalies in the corrected panel; Figure S6. Monthly outcome definitions over the complete 96-month calendar skeleton; Figure S7. Lagged national concordance between notified cases and dengue-associated deaths; Figure S8. Leave-one-component-out sensitivity of the broad ICD-only syndromic outcome; Figure S9. Diagnostics across the corrected primary and sensitivity models; Figure S10. Sensitivity of the fixed-province geographic classification; Figure S11. Full-year 2024 outcome reconciliation by month; Figure S12. Available certification characteristics for home and health-facility deaths.

## Abbreviations

CDC/MINSA	National Center for Epidemiology, Prevention and Disease Control/Ministry of Health of Peru
CI	confidence interval
DAG	directed acyclic graph
GADM	Database of Global Administrative Areas
ICD-10	International Classification of Diseases, Tenth Revision
INEI	National Institute of Statistics and Informatics
IQR	interquartile range
MRR	mortality rate ratio
NASA POWER	National Aeronautics and Space Administration Prediction of Worldwide Energy Resources
SINADEF	National Death Information System
STROBE	Strengthening the Reporting of Observational Studies in Epidemiology
