# Supplementary material for: Dengue-Associated Mortality, Climate Anomalies, and Geographic Emergence in Peru, 2017–2024: A Spatiotemporal Study Triangulating Death Records and Epidemiological Surveillance
Source: Viruses. 2026 Sep 22;18(10):1051. doi: 10.3390/v18101051 (PMC13709438; doi:10.3390/v18101051)
Supplement: Supplementary file 1 [file viruses-18-01051-s001.zip › viruses-4559758-supplementary.pdf]

Supplementary Materials

Dengue-Associated Mortality, Climate Anomalies, and Geographic Emergence in Peru, 2017–2024

Revised supplementary material following peer review

Contents

- Tables S1–S19: reporting checklist, data provenance, definitions, descriptive results, model estimates, sensitivities, diagnostics, and reproducibility QA.
- Figures S1–S12: corrected flow, conceptual framework, coding density, panel construction, climate correlations, outcome series, surveillance concordance, syndromic sensitivities, diagnostics, territorial sensitivity, full-year 2024, and home-versus-facility certification patterns.
- Separate files: high-resolution PNG/PDF figures, approved reproducibility package, and supplementary source-data archive.

Supplementary Table S1. STROBE checklist.

STROBE item	Domain	Reporting requirement	Compliance in revised manuscript
1a	Title and abstract	Identify the study design in the title or abstract using a commonly used term.	Reported in the title and abstract: retrospective ecological spatiotemporal study.
1b	Title and abstract	Provide in the abstract an informative and balanced summary of background, objective, methods, results, and conclusions.	The abstract reports the rationale, objective, data sources, primary outcome, unit, model, principal estimates, sensitivity findings, and limitations of ecological interpretation.
2	Introduction	Explain the scientific background and rationale for the study.	The Introduction describes dengue burden, climate-sensitive transmission, mortality-record evidence, and the rationale for multisource triangulation.
3	Introduction	State specific objectives, including pre-specified hypotheses when applicable.	The final paragraph of the Introduction states the four study objectives.
4	Methods	Present the key elements of the study design at the beginning of the Methods section.	The design is identified in Methods, Section 2.1.
5	Methods	Describe the setting, locations, relevant dates, and periods of recruitment, exposure, follow-up, and data collection.	The Peruvian national setting, 2017–2024 period, and fixed 196-province framework are reported in Methods.
6a	Methods	Indicate eligibility criteria and methods for selecting participants or study units.	Eligibility, geographic exclusions, and analytical stages are defined in Methods and Figure S1.
6b	Methods	For matched studies, indicate matching criteria and numbers of exposed/unexposed participants.	Not applicable; no matched case-control design was used.
7	Methods	Clearly define outcomes, exposures, predictors, confounders, effect modifiers, and diagnostic criteria.	Outcomes, exposures, covariates, geographic categories, and sensitivity definitions are specified in Methods and Tables S3–S4.
8	Methods	Describe data sources and measurement methods for each variable; when there is more than one group, report method comparability.	Data sources and measurement procedures are reported in Methods, Table S2, and the reproducibility archive.
9	Methods	Describe efforts to address potential sources of bias.	Potential ascertainment, ecological, temporal, geographic, and certification biases are addressed in Methods and Limitations.
10	Methods	Explain how the study size was determined.	No sample-size calculation was used; all eligible national records and all province-month cells were analyzed.
11	Methods	Explain how quantitative variables were handled and, where applicable, the choice of categories.	Quantitative variables, climate transformations, percentile contrasts, population denominators, and category thresholds are defined in Methods and Table S10.
12a	Methods	Describe all statistical methods, including those used to control confounding.	All statistical methods are reported in Methods, including the population offset, province fixed effects, temporal terms, lag construction, and clustered variance.
12b	Methods	Describe methods used to examine subgroups and interactions.	Subgroup/effect-modification analyses from the earlier draft were removed; the revised article reports only the approved closed sensitivity family.
12c	Methods	Explain how missing data were handled.	Geographic and climate missingness, unpopulated cause positions, and left-join rules are reported in Methods, Figure S1, and Table S7.
12d	Methods	Where applicable, explain methods specific to cohort, case-control, or complex cross-sectional survey designs.	Sensitivity analyses are reported in Methods and Tables S12–S15.
12e	Methods	Describe sensitivity analyses.	Not applicable; no sampling weights were used.
13a	Results	Report the number of units at each stage of the study.	Numbers at each analytical stage are reported in Results and Figure S1.
13b	Results	Indicate reasons for non-participation or exclusion at each stage.	Reasons for geographic exclusion, lag loss, and fixed-effect province retention are reported separately.
13c	Results	Consider using a flow diagram.	The corrected flow diagram is provided as Figure S1.
14a	Results	Present characteristics of participants or study units and the distribution of potential confounders.	Certificate characteristics are reported in Table 2 and Table S12b.
14b	Results	Indicate the number of observations with missing data for each variable of interest.	Unpopulated A–F positions are distinguished from conventional missingness in Table S5.
14c	Results	For cohort studies, summarize follow-up time.	The article reports 2018 population carried back to 2017; no follow-up time summary is applicable.
15	Results	Report the number of outcome events or summary measures.	Annual/monthly and geographic outcome counts and population rates are reported in Results and Tables S6–S6b.
16a	Results	Present unadjusted and, where applicable, confounder-adjusted estimates with statistical precision.	MRRs with 95% confidence intervals are reported for the primary and sensitivity models.
16b	Results	Report category boundaries when continuous variables are categorized.	All category boundaries and climate q90-versus-q50 contrasts are explicitly defined.
16c	Results	When relevant, translate relative estimates into absolute risk for a meaningful period.	Absolute risks are not derived from ecological MRRs; descriptive national rates are reported separately.
17	Results	Report other analyses performed, such as subgroup, interaction, and sensitivity analyses.	All reviewer-requested outcome and temporal sensitivities are reported in Tables S12–S14 and Figures 4/S8.
18	Discussion	Summarize the main findings in relation to the study objectives.	The Discussion summarizes the principal updated findings relative

			to the objectives.
19	Discussion	Discuss limitations, including the potential direction and magnitude of bias or imprecision.	Limitations address outcome classification, coding density, home deaths, ecological inference, pandemic-period control, linkage, climate assignment, population approximation, and source provenance.
20	Discussion	Provide a cautious interpretation considering objectives, limitations, multiplicity, similar studies, and relevant evidence.	Results are interpreted cautiously in light of ecological design, multiple-cause certification, consistency with prior evidence, and sensitivity analyses.
21	Discussion	Discuss generalizability or external validity of the results.	The Discussion addresses national and international analytical and public-health relevance without claiming intervention effectiveness.
22	Other information	Indicate the funding source and the role of funders.	Funding is reported in the Funding statement.

STROBE = Strengthening the Reporting of Observational Studies in Epidemiology.

Supplementary Table S2. Data sources, role, temporal coverage, and provenance limitations.

Source	Analytical role	Coverage	Corrected implementation	Limitation
SINADEF	Death certificates; codes/text in positions A–F; demographic and place-of-death variables	2017–2024	One updated snapshot used for the full period	Exact official download date and certificate-level registration/update timestamps were not preserved; administrative exhaustiveness cannot be certified.
CDC/MINSA dengue surveillance	Notified cases and severe cases	2017–2024	772,387 cases and 2,775 severe cases after canonical province mapping	No nominal linkage to SINADEF; aggregated triangulation only.
INEI population projections	Annual provincial population denominators	2018–2024; 2018 carried back to 2017	Official 196-province framework and denominators	2017 population is an operational approximation.
NASA POWER	Monthly mean temperature and precipitation for provincial centroids; 1991–2020 climatology	1991–2024	Complete coverage for 196 provinces after correction	Centroids do not represent intraprovincial microclimates.
INEI UBIGEO / GADM	Canonical names/codes and auxiliary geometry	Fixed analytical framework	INEI authoritative; GADM auxiliary only	Geographic emergence refers to fixed provinces, not boundary change.

Supplementary Table S3. Analytical dictionary and recoding.

variable	definition	type
source_id	Certificate identifier within the updated extract	string
death_date	Date of death	date
province_id	Canonical four-digit INEI province code	string
territorial_eligible	Peru residence and canonical province/district match	boolean
outcome_any_a90_a91	A90/A91 in any A-F position	boolean
outcome_position_a	A90/A91 in position A; not underlying cause	boolean
outcome_a90_only	A90 present and A91 absent	boolean
outcome_a91_only	A91 present and A90 absent	boolean
outcome_a90_a91_both	Both A90 and A91 present	boolean
outcome_syndromic_broad_icd_only	Any of six frozen syndrome ICD groups in A-F; text excluded	boolean
outcome_syndromic_narrow_icd_only	Shock or hemorrhage/coagulopathy ICD group in A-F; text excluded	boolean
outcome_syndromic_text_augmented_exploratory	Broad ICD-only or frozen term on a non-A90/A91 line; exploratory only	boolean
syndrome_text_only_exploratory	Exploratory text positive with no broad ICD component	boolean
autopsy_recorded_yes	Exact normalized value equals SI SE REALIZO NECROPSIA	boolean
n_icd10_codes	Number of populated A-F code positions	integer
first_dengue_position	Earliest A-F position containing A90/A91	category
population	INEI annual provincial population; 2017 carried back from 2018	float
temp_anom	Temperature minus 1991-2020 province-month mean	float
precip_anom	log1p precipitation minus 1991-2020 province-month mean	float
primary_model_eligible	Population and climate lags 0-3 available	boolean
surveillance_cases	CDC/MINSA notified cases after complete canonical mapping; not an eligibility criterion	integer

Supplementary Table S4. Outcome definitions and exact ICD-10 syndromic code groups.

outcome	algorithm	certificates
Any A90/A91	A90 or A91 in any A-F position	987
A90 only	A90 in A-F and no A91	378
A91 only	A91 in A-F and no A90	603
Both A90 and A91	Both code families on one certificate	6
Position A	A90/A91 in position A; not labeled underlying cause	178
Syndromic broad ICD-only	A90/A91 plus >=1 ICD prefix from any of six frozen clinical groups	761
Syndromic narrow ICD-only	A90/A91 plus an ICD prefix for shock or hemorrhage/coagulopathy/thrombocytopenia	409
Syndromic text-augmented exploratory	Broad ICD-only or a frozen text term on a line not paired with A90/A91	776
Text-only exploratory contribution	Text-augmented positive without any broad ICD component	15

Exact ICD-10 prefix dictionary for the broad and narrow sensitivity outcomes

syndrome_group	icd10_prefixes	broad_membership	narrow_membership	clinical_rationale
hemorrhage	D65, D66, D67, D68, D69, K92, R58	yes	yes	Hemorrhage/coagulopathy or thrombocytopenia compatible with severe dengue
hepatic_failure	K70, K71, K72, K73, K74, K75, K76, K77	yes	no	Organ failure compatible with severe illness; broad liver codes reduce specificity
renal_failure	N17, N18, N19	yes	no	Organ failure compatible with severe illness; chronic codes reduce specificity
respiratory_failure	J80, J81, J96	yes	no	Severe clinical state but etiologically nonspecific
sepsis	A40, A41	yes	no	Sepsis is clinically severe but etiologically nonspecific
shock	R57	yes	yes	Hemodynamic collapse compatible with severe dengue but not etiologically specific

The broad and narrow model outcomes use ICD-10 prefixes only. Frozen text terms were evaluated only as an exploratory sensitivity and excluded the text line paired with A90/A91. No dated evidence was available to establish prespecification before outcome inspection.

Supplementary Table S5. ICD-10 position population and coding depth by year.

Year	A90/A91 certificates	Median positions	P25	P75	A90/A91 in position A (%)	First A90/A91 in B–F (%)	Invalid code (%)	at_least_2_positions_percent	at_least_3_positions_percent	at_least_4_positions_percent	at_least_5_positions_percent	at_least_6_positions_percent	All deaths: A populated (%)	All deaths: B populated (%)	All deaths: C populated (%)	All deaths: D populated (%)	All deaths: E populated (%)	All deaths: F populated (%)
2,017	64	3	2	4	14.062	85.938	0	95.312	71.875	42.188	14.062	4.688	75.743	60.611	40.569	17.595	12.086	3.678
2,018	16	3	2	3	18.75	81.25	0	93.75	56.25	18.75	0	0	72.214	59.114	39.634	16.23	12.689	4.118
2,019	27	3	2	4	25.926	74.074	0	88.889	66.667	29.63	7.407	0	72.832	59.802	39.957	15.86	13.396	4.264
2,020	131	3	2	4	22.137	77.863	0	89.313	70.229	36.641	11.45	3.053	81.409	69.534	44.656	13.266	14.326	4.444
2,021	116	3	2.75	4	12.069	87.931	0	93.966	75	43.103	15.517	2.586	84.04	72.404	47.961	12.748	14.508	4.552
2,022	134	3	3	4	17.164	82.836	0	94.03	77.612	44.776	14.925	5.97	81.392	66.477	42.557	14.107	14.099	4.564
2,023	376	3	2	4	19.947	80.053	0	89.096	72.074	39.628	11.17	2.926	85.392	67.658	41.78	14.421	11.775	3.532
2,024	123	3	3	4	14.634	85.366	0	91.87	75.61	45.528	15.447	7.317	85.05	69.506	42.522	13.937	13.027	3.962

Blank later cause positions are reported as unpopulated, not automatically as missing, because a valid certificate need not contain six conditions.

Supplementary Table S6a. Annual territorially eligible deaths, population, and mortality rates.

Year	Deaths	National population	Rate per 100,000
2,017	60	31,562,130	0.19
2,018	16	31,562,130	0.051
2,019	27	32,131,400	0.084
2,020	130	32,625,948	0.398
2,021	116	33,035,304	0.351
2,022	134	33,396,698	0.401
2,023	363	33,725,844	1.076
2,024	122	34,038,457	0.358

Rates use the sum of province-specific populations. They do not divide national deaths by a mean provincial denominator.

Supplementary Table S6b. Department-year dengue-associated mortality and population rates.

Department	Year	Deaths	Population	Rate per 100,000
AMAZONAS	2,017	0	419,833	0
AMAZONAS	2,018	0	419,833	0
AMAZONAS	2,019	0	423,863	0
AMAZONAS	2,020	2	426,806	0.469
AMAZONAS	2,021	8	428,512	1.867
AMAZONAS	2,022	8	429,483	1.863
AMAZONAS	2,023	0	429,943	0
AMAZONAS	2,024	0	430,123	0
ANCASH	2,017	0	1,155,451	0
ANCASH	2,018	0	1,155,451	0
ANCASH	2,019	0	1,169,522	0
ANCASH	2,020	1	1,180,638	0.085
ANCASH	2,021	0	1,188,391	0
ANCASH	2,022	0	1,194,156	0
ANCASH	2,023	7	1,198,547	0.584
ANCASH	2,024	10	1,202,171	0.832
APURIMAC	2,017	0	427,323	0
APURIMAC	2,018	0	427,323	0
APURIMAC	2,019	0	429,587	0
APURIMAC	2,020	0	430,736	0
APURIMAC	2,021	0	430,609	0
APURIMAC	2,022	0	429,720	0
APURIMAC	2,023	0	428,311	0
APURIMAC	2,024	0	426,626	0
AREQUIPA	2,017	0	1,428,708	0
AREQUIPA	2,018	0	1,428,708	0
AREQUIPA	2,019	0	1,464,638	0
AREQUIPA	2,020	0	1,497,438	0
AREQUIPA	2,021	0	1,526,669	0
AREQUIPA	2,022	0	1,553,994	0
AREQUIPA	2,023	0	1,580,075	0
AREQUIPA	2,024	0	1,605,569	0
AYACUCHO	2,017	0	659,061	0
AYACUCHO	2,018	0	659,061	0
AYACUCHO	2,019	0	664,494	0
AYACUCHO	2,020	4	668,213	0.599
AYACUCHO	2,021	1	669,979	0.149
AYACUCHO	2,022	1	670,579	0.149
AYACUCHO	2,023	1	670,377	0.149
AYACUCHO	2,024	1	669,737	0.149
CAJAMARCA	2,017	0	1,438,325	0
CAJAMARCA	2,018	1	1,438,325	0.07
CAJAMARCA	2,019	0	1,447,891	0
CAJAMARCA	2,020	2	1,453,711	0.138
CAJAMARCA	2,021	2	1,455,245	0.137
CAJAMARCA	2,022	7	1,454,217	0.481
CAJAMARCA	2,023	2	1,451,436	0.138
CAJAMARCA	2,024	3	1,447,707	0.207
CALLAO	2,017	0	1,078,789	0
CALLAO	2,018	0	1,078,789	0
CALLAO	2,019	0	1,105,512	0
CALLAO	2,020	0	1,129,854	0
CALLAO	2,021	0	1,151,480	0
CALLAO	2,022	0	1,171,648	0
CALLAO	2,023	1	1,190,860	0.084
CALLAO	2,024	1	1,209,614	0.083
CUSCO	2,017	0	1,320,530	0
CUSCO	2,018	0	1,320,530	0
CUSCO	2,019	0	1,340,457	0
CUSCO	2,020	4	1,357,075	0.295

CUSCO	2,021	1	1,369,932	0.073
CUSCO	2,022	1	1,380,594	0.072
CUSCO	2,023	2	1,389,737	0.144
CUSCO	2,024	1	1,398,036	0.072
HUANCAVELICA	2,017	0	376,346	0
HUANCAVELICA	2,018	0	376,346	0
HUANCAVELICA	2,019	0	371,260	0
HUANCAVELICA	2,020	0	365,317	0
HUANCAVELICA	2,021	0	358,356	0
HUANCAVELICA	2,022	0	350,845	0
HUANCAVELICA	2,023	2	343,026	0.583
HUANCAVELICA	2,024	1	335,142	0.298
HUANUCO	2,017	0	757,467	0
HUANUCO	2,018	0	757,467	0
HUANUCO	2,019	0	759,851	0
HUANUCO	2,020	1	760,267	0.132
HUANUCO	2,021	5	758,416	0.659
HUANUCO	2,022	2	755,213	0.265
HUANUCO	2,023	0	751,097	0
HUANUCO	2,024	0	746,509	0
ICA	2,017	3	923,175	0.325
ICA	2,018	0	923,175	0
ICA	2,019	0	950,100	0
ICA	2,020	8	975,182	0.82
ICA	2,021	8	998,144	0.801
ICA	2,022	6	1,020,050	0.588
ICA	2,023	45	1,041,312	4.321
ICA	2,024	29	1,062,346	2.73
JUNIN	2,017	2	1,335,139	0.15
JUNIN	2,018	0	1,335,139	0
JUNIN	2,019	1	1,350,021	0.074
JUNIN	2,020	13	1,361,467	0.955
JUNIN	2,021	20	1,369,003	1.461
JUNIN	2,022	8	1,374,221	0.582
JUNIN	2,023	5	1,377,838	0.363
JUNIN	2,024	3	1,380,561	0.217
LA LIBERTAD	2,017	2	1,938,501	0.103
LA LIBERTAD	2,018	0	1,938,501	0
LA LIBERTAD	2,019	0	1,979,901	0
LA LIBERTAD	2,020	1	2,016,771	0.05
LA LIBERTAD	2,021	1	2,048,492	0.049
LA LIBERTAD	2,022	2	2,077,345	0.096
LA LIBERTAD	2,023	22	2,104,254	1.046
LA LIBERTAD	2,024	19	2,130,145	0.892
LAMBAYEQUE	2,017	5	1,270,295	0.394
LAMBAYEQUE	2,018	0	1,270,295	0
LAMBAYEQUE	2,019	1	1,292,105	0.077
LAMBAYEQUE	2,020	4	1,310,785	0.305
LAMBAYEQUE	2,021	1	1,325,912	0.075
LAMBAYEQUE	2,022	1	1,338,994	0.075
LAMBAYEQUE	2,023	92	1,350,663	6.811
LAMBAYEQUE	2,024	9	1,361,554	0.661
LIMA	2,017	2	10,180,641	0.02
LIMA	2,018	1	10,180,641	0.01
LIMA	2,019	1	10,416,139	0.01
LIMA	2,020	3	10,628,470	0.028
LIMA	2,021	3	10,814,450	0.028
LIMA	2,022	0	10,986,006	0
LIMA	2,023	15	11,147,924	0.135
LIMA	2,024	23	11,304,993	0.203
LORETO	2,017	1	1,000,350	0.1
LORETO	2,018	1	1,000,350	0.1
LORETO	2,019	2	1,015,212	0.197

LORETO	2,020	23	1,027,559	2.238
LORETO	2,021	11	1,037,055	1.061
LORETO	2,022	9	1,044,884	0.861
LORETO	2,023	7	1,051,560	0.666
LORETO	2,024	4	1,057,592	0.378
MADRE DE DIOS	2,017	7	161,324	4.339
MADRE DE DIOS	2,018	8	161,324	4.959
MADRE DE DIOS	2,019	17	167,674	10.139
MADRE DE DIOS	2,020	26	173,811	14.959
MADRE DE DIOS	2,021	12	179,688	6.678
MADRE DE DIOS	2,022	30	185,478	16.174
MADRE DE DIOS	2,023	2	191,259	1.046
MADRE DE DIOS	2,024	1	197,096	0.507
MOQUEGUA	2,017	0	186,371	0
MOQUEGUA	2,018	0	186,371	0
MOQUEGUA	2,019	0	189,781	0
MOQUEGUA	2,020	0	192,740	0
MOQUEGUA	2,021	0	195,185	0
MOQUEGUA	2,022	0	197,337	0
MOQUEGUA	2,023	0	199,286	0
MOQUEGUA	2,024	0	201,129	0
PASCO	2,017	0	271,704	0
PASCO	2,018	0	271,704	0
PASCO	2,019	0	272,157	0
PASCO	2,020	1	271,904	0.368
PASCO	2,021	2	270,842	0.738
PASCO	2,022	2	269,296	0.743
PASCO	2,023	0	267,425	0
PASCO	2,024	0	265,392	0
PIURA	2,017	35	1,974,368	1.773
PIURA	2,018	0	1,974,368	0
PIURA	2,019	0	2,013,517	0
PIURA	2,020	1	2,047,954	0.049
PIURA	2,021	4	2,077,039	0.193
PIURA	2,022	33	2,103,099	1.569
PIURA	2,023	133	2,127,093	6.253
PIURA	2,024	16	2,149,978	0.744
PUNO	2,017	0	1,236,836	0
PUNO	2,018	1	1,236,836	0.081
PUNO	2,019	0	1,239,022	0
PUNO	2,020	0	1,237,997	0
PUNO	2,021	0	1,233,277	0
PUNO	2,022	2	1,226,353	0.163
PUNO	2,023	0	1,217,951	0
PUNO	2,024	0	1,208,802	0
SAN MARTIN	2,017	0	866,861	0
SAN MARTIN	2,018	1	866,861	0.115
SAN MARTIN	2,019	4	884,283	0.452
SAN MARTIN	2,020	9	899,648	1
SAN MARTIN	2,021	23	912,674	2.52
SAN MARTIN	2,022	13	924,384	1.406
SAN MARTIN	2,023	8	935,194	0.855
SAN MARTIN	2,024	0	945,523	0
TACNA	2,017	0	354,644	0
TACNA	2,018	0	354,644	0
TACNA	2,019	0	363,205	0
TACNA	2,020	0	370,974	0
TACNA	2,021	1	377,842	0.265
TACNA	2,022	0	384,222	0
TACNA	2,023	0	390,279	0
TACNA	2,024	0	396,180	0
TUMBES	2,017	3	241,321	1.243
TUMBES	2,018	0	241,321	0

TUMBES	2,019	0	246,699	0
TUMBES	2,020	10	251,521	3.976
TUMBES	2,021	8	255,712	3.129
TUMBES	2,022	0	259,556	0
TUMBES	2,023	14	263,164	5.32
TUMBES	2,024	0	266,653	0
UCAYALI	2,017	0	558,767	0
UCAYALI	2,018	3	558,767	0.537
UCAYALI	2,019	1	574,509	0.174
UCAYALI	2,020	17	589,110	2.886
UCAYALI	2,021	5	602,400	0.83
UCAYALI	2,022	9	615,024	1.463
UCAYALI	2,023	5	627,233	0.797
UCAYALI	2,024	1	639,279	0.156

Supplementary Table S7. Corrected analytical flow and canonical unit counts.

stage	certificates_or_deaths	province_month_cells	note
R1 updated source records 2017-2024	1,285,955		Single updated vintage
R1 A90/A91 certificates	987		Any position A-F
R1 residence/canonical geography eligible	968		Province and district matched to INEI
R1 represented in balanced panel	968	18,816	196 x 96; excluded certificates retained in lineage
R1 population available	968	18,816	Population is not zero-filled
R1 climate lags 0-3 available	959	18,228	First 3 months/province unavailable by design

Inherited and corrected unit reconciliation

unit	cells	interpretation
R0 province-month skeleton	17,424	198 x 88; noncanonical
R0 climate rows described	11,704	133 x 88
R0 model sample	11,220	132 provinces
R1 province-month skeleton	18,816	196 x 96
R1 lag-eligible candidate	18,228	196 x 93 after 3 lag months

Supplementary Table S8. Climate-exposure definitions, contrasts, and descriptive statistics.

parameter	value
lag_basis	unconstrained separate coefficients at lags 0,1,2,3
anomaly	observed minus province-calendar-month 1991-2020 mean; precipitation uses log1p before subtraction
standardization	global mean/SD over corrected 2017-2024 province-month panel, matching inherited code rather than climatological SD
contrast	unweighted q90-q50 of corrected main lag-eligible panel, fixed across R1 outcome sensitivities
q_values.temp_q50	-0.06399318401455711
q_values.temp_q90	1.340264386048873
q_values.precip_q50	-0.06241642011989376
q_values.precip_q90	1.1231197837274738

Descriptive distribution in the 196-province by 96-month panel

Exposure	count	mean	std	min	10%	25%	50%	75%	90%	max
temp_anom_z	18,816	-6.04e-17	1	-4.17	-1.154	-0.667	-0.088	0.547	1.32	5.758
precip_anom_z	18,816	-3.02e-17	1	-4.245	-1.16	-0.513	-0.049	0.482	1.176	8.15
humidity_anom_z	18,816	2.42e-17	1	-6.418	-1.145	-0.511	0.064	0.573	1.142	4.566

Supplementary Table S9a. Summary of aggregated SINADEF–surveillance concordance.

Metric	Estimate	Scope
Notified dengue cases	772,387	Canonical 2017–2024 province-month panel
Notified severe cases	2,775	Canonical 2017–2024 province-month panel
Territorially eligible deaths	968	Balanced panel
Department-year Spearman ρ	0.772	p = 8.349e-41; 200 department-years
Peak-month difference, median (IQR)	0 (-2 to 1)	102 department-years with both deaths and cases

Supplementary Table S9b. Department-year mortality and epidemiological-surveillance counts.

Department	Year	Deaths	Notified cases	Severe cases	Deaths per 1,000 cases
AMAZONAS	2,017	0	93	0	0
AMAZONAS	2,018	0	109	0	0
AMAZONAS	2,019	0	165	0	0
AMAZONAS	2,020	2	872	3	2.294
AMAZONAS	2,021	8	2,369	3	3.377
AMAZONAS	2,022	8	3,575	8	2.238
AMAZONAS	2,023	0	3,271	8	0
AMAZONAS	2,024	0	1,517	2	0
ANCASH	2,017	0	1,720	5	0
ANCASH	2,018	0	6	1	0
ANCASH	2,019	0	20	0	0
ANCASH	2,020	1	0	0	
ANCASH	2,021	0	28	1	0
ANCASH	2,022	0	2,145	11	0
ANCASH	2,023	7	11,658	14	0.6
ANCASH	2,024	10	18,789	52	0.532
APURIMAC	2,017	0	0	0	
APURIMAC	2,018	0	0	0	
APURIMAC	2,019	0	0	0	
APURIMAC	2,020	0	0	0	
APURIMAC	2,021	0	0	0	
APURIMAC	2,022	0	0	0	
APURIMAC	2,023	0	0	0	
APURIMAC	2,024	0	11	0	0
AREQUIPA	2,017	0	0	0	
AREQUIPA	2,018	0	0	0	
AREQUIPA	2,019	0	0	0	
AREQUIPA	2,020	0	0	0	
AREQUIPA	2,021	0	7	0	0
AREQUIPA	2,022	0	0	0	
AREQUIPA	2,023	0	0	0	
AREQUIPA	2,024	0	21	0	0
AYACUCHO	2,017	0	1,657	6	0
AYACUCHO	2,018	0	202	0	0
AYACUCHO	2,019	0	95	1	0
AYACUCHO	2,020	4	1,583	4	2.527
AYACUCHO	2,021	1	1,663	1	0.601
AYACUCHO	2,022	1	782	0	1.279
AYACUCHO	2,023	1	695	2	1.439
AYACUCHO	2,024	1	362	0	2.762
CAJAMARCA	2,017	0	420	0	0
CAJAMARCA	2,018	1	7	0	142.857
CAJAMARCA	2,019	0	397	0	0
CAJAMARCA	2,020	2	266	0	7.519
CAJAMARCA	2,021	2	4,015	8	0.498
CAJAMARCA	2,022	7	3,639	2	1.924
CAJAMARCA	2,023	2	7,451	8	0.268
CAJAMARCA	2,024	3	2,850	8	1.053
CALLAO	2,017	0	5	0	0
CALLAO	2,018	0	0	0	
CALLAO	2,019	0	0	0	
CALLAO	2,020	0	4	0	0
CALLAO	2,021	0	9	0	0
CALLAO	2,022	0	8	0	0

Department	Year	Deaths	Notified cases	Severe cases	Deaths per 1,000 cases
CALLAO	2,023	1	2,356	10	0.424
CALLAO	2,024	1	5,756	11	0.174
CUSCO	2,017	0	537	8	0
CUSCO	2,018	0	79	2	0
CUSCO	2,019	0	66	2	0
CUSCO	2,020	4	2,487	3	1.608
CUSCO	2,021	1	1,982	1	0.505
CUSCO	2,022	1	3,687	3	0.271
CUSCO	2,023	2	2,630	8	0.76
CUSCO	2,024	1	2,104	7	0.475
HUANCAVELICA	2,017	0	0	0	
HUANCAVELICA	2,018	0	0	0	
HUANCAVELICA	2,019	0	0	0	
HUANCAVELICA	2,020	0	0	0	
HUANCAVELICA	2,021	0	0	0	
HUANCAVELICA	2,022	0	0	0	
HUANCAVELICA	2,023	2	0	0	
HUANCAVELICA	2,024	1	0	0	
HUANUCO	2,017	0	92	0	0
HUANUCO	2,018	0	26	1	0
HUANUCO	2,019	0	34	0	0
HUANUCO	2,020	1	1,133	2	0.883
HUANUCO	2,021	5	2,811	11	1.779
HUANUCO	2,022	2	1,580	8	1.266
HUANUCO	2,023	0	2,298	6	0
HUANUCO	2,024	0	775	0	0
ICA	2,017	3	4,384	8	0.684
ICA	2,018	0	127	0	0
ICA	2,019	0	51	0	0
ICA	2,020	8	7,144	8	1.12
ICA	2,021	8	4,208	6	1.901
ICA	2,022	6	5,084	10	1.18
ICA	2,023	45	16,889	68	2.664
ICA	2,024	29	30,407	79	0.954
JUNIN	2,017	2	220	1	9.091
JUNIN	2,018	0	53	1	0
JUNIN	2,019	1	504	3	1.984
JUNIN	2,020	13	3,714	12	3.5
JUNIN	2,021	20	5,090	12	3.929
JUNIN	2,022	8	4,157	11	1.924
JUNIN	2,023	5	3,580	24	1.397
JUNIN	2,024	3	3,331	10	0.901
LA LIBERTAD	2,017	2	5,904	10	0.339
LA LIBERTAD	2,018	0	3	2	0
LA LIBERTAD	2,019	0	366	1	0
LA LIBERTAD	2,020	1	390	0	2.564
LA LIBERTAD	2,021	1	262	1	3.817
LA LIBERTAD	2,022	2	162	0	12.346
LA LIBERTAD	2,023	22	26,502	87	0.83
LA LIBERTAD	2,024	19	49,931	119	0.381
LAMBAYEQUE	2,017	5	1,579	6	3.167
LAMBAYEQUE	2,018	0	4	0	0
LAMBAYEQUE	2,019	1	770	1	1.299
LAMBAYEQUE	2,020	4	568	13	7.042
LAMBAYEQUE	2,021	1	843	1	1.186
LAMBAYEQUE	2,022	1	2,386	1	0.419
LAMBAYEQUE	2,023	92	31,460	240	2.924
LAMBAYEQUE	2,024	9	9,305	45	0.967
LIMA	2,017	2	362	0	5.525

Department	Year	Deaths	Notified cases	Severe cases	Deaths per 1,000 cases
LIMA	2,018	1	11	0	90.909
LIMA	2,019	1	46	0	21.739
LIMA	2,020	3	350	1	8.571
LIMA	2,021	3	1,190	0	2.521
LIMA	2,022	0	938	2	0
LIMA	2,023	15	30,735	76	0.488
LIMA	2,024	23	87,864	208	0.262
LORETO	2,017	1	1,089	13	0.918
LORETO	2,018	1	1,984	27	0.504
LORETO	2,019	2	2,579	28	0.775
LORETO	2,020	23	7,617	56	3.02
LORETO	2,021	11	5,115	22	2.151
LORETO	2,022	9	8,926	32	1.008
LORETO	2,023	7	6,582	13	1.064
LORETO	2,024	4	7,996	10	0.5
MADRE DE DIOS	2,017	7	565	11	12.389
MADRE DE DIOS	2,018	8	1,249	22	6.405
MADRE DE DIOS	2,019	17	7,605	103	2.235
MADRE DE DIOS	2,020	26	3,011	24	8.635
MADRE DE DIOS	2,021	12	1,333	10	9.002
MADRE DE DIOS	2,022	30	3,641	20	8.239
MADRE DE DIOS	2,023	2	1,853	9	1.079
MADRE DE DIOS	2,024	1	505	3	1.98
MOQUEGUA	2,017	0	0	0	
MOQUEGUA	2,018	0	1	0	0
MOQUEGUA	2,019	0	0	0	
MOQUEGUA	2,020	0	0	0	
MOQUEGUA	2,021	0	0	0	
MOQUEGUA	2,022	0	0	0	
MOQUEGUA	2,023	0	0	0	
MOQUEGUA	2,024	0	0	0	
PASCO	2,017	0	4	0	0
PASCO	2,018	0	3	0	0
PASCO	2,019	0	39	0	0
PASCO	2,020	1	374	10	2.674
PASCO	2,021	2	616	2	3.247
PASCO	2,022	2	496	2	4.032
PASCO	2,023	0	1,156	3	0
PASCO	2,024	0	683	2	0
PIURA	2,017	35	44,275	141	0.791
PIURA	2,018	0	525	1	0
PIURA	2,019	0	70	1	0
PIURA	2,020	1	125	2	8
PIURA	2,021	4	4,072	10	0.982
PIURA	2,022	33	12,151	51	2.716
PIURA	2,023	133	79,304	311	1.677
PIURA	2,024	16	32,246	141	0.496
PUNO	2,017	0	0	0	
PUNO	2,018	1	0	0	
PUNO	2,019	0	68	0	0
PUNO	2,020	0	17	0	0
PUNO	2,021	0	51	0	0
PUNO	2,022	2	25	0	80
PUNO	2,023	0	267	0	0
PUNO	2,024	0	201	0	0
SAN MARTIN	2,017	0	460	0	0
SAN MARTIN	2,018	1	100	0	10
SAN MARTIN	2,019	4	2,063	16	1.939
SAN MARTIN	2,020	9	4,859	31	1.852

**Department**	**Year**	**Deaths**	**Notified cases**	**Severe cases**	**Deaths per 1,000 cases**
SAN MARTIN	2,021	23	4,532	15	5.075
SAN MARTIN	2,022	13	4,270	24	3.044
SAN MARTIN	2,023	8	6,890	30	1.161
SAN MARTIN	2,024	0	10,870	36	0
TACNA	2,017	0	0	0	
TACNA	2,018	0	0	0	
TACNA	2,019	0	0	0	
TACNA	2,020	0	0	0	
TACNA	2,021	1	0	0	
TACNA	2,022	0	0	0	
TACNA	2,023	0	0	0	
TACNA	2,024	0	0	0	
TUMBES	2,017	3	4,145	7	0.724
TUMBES	2,018	0	65	0	0
TUMBES	2,019	0	524	0	0
TUMBES	2,020	10	3,076	1	3.251
TUMBES	2,021	8	1,455	3	5.498
TUMBES	2,022	0	723	0	0
TUMBES	2,023	14	12,890	62	1.086
TUMBES	2,024	0	5,267	2	0
UCAYALI	2,017	0	779	18	0
UCAYALI	2,018	3	319	8	9.404
UCAYALI	2,019	1	220	6	4.545
UCAYALI	2,020	17	9,772	50	1.74
UCAYALI	2,021	5	3,140	22	1.592
UCAYALI	2,022	9	4,842	27	1.859
UCAYALI	2,023	5	8,174	38	0.612
UCAYALI	2,024	1	740	1	1.351

Supplementary Table S10. Complete computational specification of the climate model.

parameter	value
software	Python statsmodels GLM
family	Poisson
link	log
offset	log annual provincial population
province_effect	indicator fixed effects; outcome-specific no-variation provinces removed and counted
seasonality	calendar-month categorical indicators
time	linear time_index from Jan 2017; original calendar retained when 2020-2021 excluded
pandemic	binary 2020-2021 except M8, where period is removed and indicator omitted
exposure_basis	linear standardized anomalies
lag_basis	unconstrained separate coefficients at lags 0,1,2,3
anomaly	observed minus province-calendar-month 1991-2020 mean; precipitation uses log1p before subtraction
standardization	global mean/SD over corrected 2017-2024 province-month panel, matching inherited code rather than climatological SD
contrast	unweighted q90-q50 of corrected main lag-eligible panel, fixed across R1 outcome sensitivities
variance	sandwich covariance clustered by province
q_values.temp_q50	-0.06399318401455711
q_values.temp_q90	1.340264386048873
q_values.precip_q50	-0.06241642011989376
q_values.precip_q90	1.1231197837274738
candidate_period	2017-2024
missing_lags	first three months per province excluded
surveillance_eligibility	not used
seed	20260901

Supplementary Table S11. Corrected primary climate-model estimates.

model_id	outcome	exposure	lag_window	used_provinces	used_cells	used_events	q50	q90	mrr	ci_low	ci_high	pearson_df	converged
M0-R1	Any A90/A91	Temperature anomaly	0-3 months	92	8,556	959	-0.064	1.34	1.749	1.412	2.168	1.481	1
M0-R1	Any A90/A91	Precipitation anomaly	0-3 months	92	8,556	959	-0.062	1.123	2.687	1.966	3.673	1.481	1

Supplementary Table S12a. Climate-model sensitivity analyses by outcome definition.

model_id	outcome	exposure	used_provinces	used_cells	used_events	mrr	ci_low	ci_high	pearson_df	stability
M1	A90/A91 in position A	Temperature anomaly	49	4,557	175	1.718	1.184	2.492	1.411	inferentially estimable
M1	A90/A91 in position A	Precipitation anomaly	49	4,557	175	3.286	1.836	5.882	1.411	inferentially estimable
M2	Any A90	Temperature anomaly	68	6,324	371	2.039	1.615	2.576	1.432	inferentially estimable
M2	Any A90	Precipitation anomaly	68	6,324	371	3.03	2.368	3.876	1.432	inferentially estimable
M3	Any A91	Temperature anomaly	77	7,161	594	1.612	1.254	2.072	1.342	inferentially estimable
M3	Any A91	Precipitation anomaly	77	7,161	594	2.435	1.645	3.605	1.342	inferentially estimable
M4	A90 only	Temperature anomaly	67	6,231	365	2.034	1.606	2.576	1.445	inferentially estimable
M4	A90 only	Precipitation anomaly	67	6,231	365	3.003	2.344	3.847	1.445	inferentially estimable
M5	A91 only	Temperature anomaly	77	7,161	588	1.608	1.249	2.07	1.339	inferentially estimable
M5	A91 only	Precipitation anomaly	77	7,161	588	2.429	1.637	3.604	1.339	inferentially estimable
M6	Broad syndromic subset, ICD-only	Temperature anomaly	87	8,091	740	1.644	1.318	2.05	1.322	inferentially estimable
M6	Broad syndromic subset, ICD-only	Precipitation anomaly	87	8,091	740	2.521	1.946	3.267	1.322	inferentially estimable
M7	Narrow shock/hemorrhagic subset, ICD-only	Temperature anomaly	75	6,975	397	1.693	1.301	2.205	1.248	inferentially estimable
M7	Narrow shock/hemorrhagic subset, ICD-only	Precipitation anomaly	75	6,975	397	2.164	1.47	3.185	1.248	inferentially estimable
M6-1	Broad ICD-only syndrome using any group except sepsis	Temperature anomaly	86	7,998	687	1.587	1.26	1.999	1.287	inferentially estimable
M6-1	Broad ICD-only syndrome using any group except sepsis	Precipitation anomaly	86	7,998	687	2.472	1.917	3.189	1.287	inferentially estimable
M6-2	Broad ICD-only syndrome using any group except respiratory failure	Temperature anomaly	81	7,533	584	1.643	1.298	2.081	1.295	inferentially estimable
M6-2	Broad ICD-only syndrome using any group except respiratory failure	Precipitation anomaly	81	7,533	584	2.416	1.759	3.319	1.295	inferentially estimable
M6-3	Broad ICD-only syndrome using any group except renal failure	Temperature anomaly	87	8,091	728	1.637	1.308	2.048	1.304	inferentially estimable
M6-3	Broad ICD-only syndrome using any group except renal failure	Precipitation anomaly	87	8,091	728	2.538	1.954	3.296	1.304	inferentially estimable
M6-4	Broad ICD-only syndrome using any group except hepatic failure	Temperature anomaly	87	8,091	727	1.652	1.321	2.065	1.318	inferentially estimable
M6-4	Broad ICD-only syndrome using any group except hepatic failure	Precipitation anomaly	87	8,091	727	2.502	1.937	3.231	1.318	inferentially estimable

Supplementary Table S12b. Home versus health-facility certification characteristics and modified-Poisson estimates.

place	n	late_position_n	late_position_percent	position_a_n	A91_any_n	syndrome_broad_icd_only_n	syndrome_narrow_icd_only_n	syndrome_text_augmented_exploratory_n	median_codes	autopsy_yes_n	institution_populated_n
Health facility	802	680	84.788	122	514	672	372	683	3	7	801
Home	143	95	66.434	48	65	60	21	62	2	0	0
Other/unknown	42	34	80.952	8	30	29	16	31	3	0	0

model	comparison	n	prevalence_ratio	ci_low	ci_high	absolute_difference_percentage_points	absolute_difference_method
Unadjusted	Home vs health facility	945	0.784	0.695	0.884	-18.354	Crude observed prevalence difference
Adjusted	Home vs health facility; adjusted for year, age group, sex	939	0.779	0.692	0.877	-18.813	Standardized marginal predictions from adjusted modified-Poisson model

The adjusted absolute difference uses standardized marginal predictions. Laboratory confirmation, prior evaluation, hospitalization, certifying-physician information, and medical-record access were unavailable.

Supplementary Table S13. Climate-model sensitivity analyses by temporal window.

model_id	outcome	period	exposure	used_provinces	used_cells	used_events	mrr	ci_low	ci_high	pearson_df	stability
M8	Any A90/A91 excluding 2020-2021	2017-2019 and 2022-2024	Temperature anomaly	85	5,865	713	1.613	1.256	2.073	1.529	inferentially estimable
M8	Any A90/A91 excluding 2020-2021	2017-2019 and 2022-2024	Precipitation anomaly	85	5,865	713	2.288	1.569	3.338	1.529	inferentially estimable
M9	Any A90/A91, complete years 2017-2023	2017-2023	Temperature anomaly	82	6,642	837	2.014	1.534	2.643	1.371	inferentially estimable
M9	Any A90/A91, complete years 2017-2023	2017-2023	Precipitation anomaly	82	6,642	837	2.342	1.744	3.146	1.371	inferentially estimable

Supplementary Table S14. Leave-one-component-out syndromic sensitivity analyses and unit tests.

model_id	outcome	exposure	used_provinces	used_cells	used_events	mrr	ci_low	ci_high	stability
M6-1	Broad ICD-only syndrome using any group except sepsis	Temperature anomaly	86	7,998	687	1.587	1.26	1.999	inferentially estimable
M6-1	Broad ICD-only syndrome using any group except sepsis	Precipitation anomaly	86	7,998	687	2.472	1.917	3.189	inferentially estimable
M6-2	Broad ICD-only syndrome using any group except respiratory failure	Temperature anomaly	81	7,533	584	1.643	1.298	2.081	inferentially estimable
M6-2	Broad ICD-only syndrome using any group except respiratory failure	Precipitation anomaly	81	7,533	584	2.416	1.759	3.319	inferentially estimable
M6-3	Broad ICD-only syndrome using any group except renal failure	Temperature anomaly	87	8,091	728	1.637	1.308	2.048	inferentially estimable
M6-3	Broad ICD-only syndrome using any group except renal failure	Precipitation anomaly	87	8,091	728	2.538	1.954	3.296	inferentially estimable
M6-4	Broad ICD-only syndrome using any group except hepatic failure	Temperature anomaly	87	8,091	727	1.652	1.321	2.065	inferentially estimable
M6-4	Broad ICD-only syndrome using any group except hepatic failure	Precipitation anomaly	87	8,091	727	2.502	1.937	3.231	inferentially estimable

Set-logic unit tests

test	observed	expected	pass
independent_recomputation_without_sepsis	705	705	1
independent_recomputation_without_respiratory_failure	600	600	1
independent_recomputation_without_renal_failure	749	749	1
independent_recomputation_without_hepatic_failure	748	748	1
unit_single removed component becomes negative	False	False	1
unit_sepsis plus shock remains positive	True	True	1
unit_three-component certificate remains positive	True	True	1
unit_unrelated component remains positive	True	True	1
legacy_combined_regression_without_sepsis	809	809	1
legacy_combined_regression_without_respiratory_failure	722	722	1
legacy_combined_regression_without_renal_failure	834	834	1
legacy_combined_regression_without_hepatic_failure	834	834	1

Supplementary Table S15. Model diagnostics and effective analytical samples.

model_id	used_cells	used_provinces	used_events	predicted_events	pearson_df	converged	warnings	outcome	period	candidate_provinces	event_provinces	candidate_cells	candidate_events	zero_cell_percent	max_province_event_share	finite_parameters	finite_covariance
M0-R0	11,220	132	529	529	0.572	1											
M0-R1	8,556	92	959	959	1.481	1		Any A90/A91	2017-2024	196	92	18,228	959	93.969	0.131	1	1
M1	4,557	49	175	175	1.411	1		A90/A91 in position A	2017-2024	196	49	18,228	175	97.103	0.149	1	1
M2	6,324	68	371	371	1.432	1		Any A90	2017-2024	196	68	18,228	371	96.3	0.148	1	1
M3	7,161	77	594	594	1.342	1		Any A91	2017-2024	196	77	18,228	594	94.875	0.138	1	1
M4	6,231	67	365	365	1.445	1		A90 only	2017-2024	196	67	18,228	365	96.293	0.148	1	1
M5	7,161	77	588	588	1.339	1		A91 only	2017-2024	196	77	18,228	588	94.889	0.139	1	1
M6	8,091	87	740	740	1.322	1		Broad syndromic subset, ICD-only	2017-2024	196	87	18,228	740	94.5	0.115	1	1
M7	6,975	75	397	397	1.248	1		Narrow shock/hemorrhagic subset, ICD-only	2017-2024	196	75	18,228	397	96.057	0.116	1	1
M8	5,865	85	713	713	1.529	1		Any A90/A91 excluding 2020-2021	2017-2019 and 2022-2024	196	85	13,524	713	94.169	0.175	1	1
M9	6,642	82	837	837	1.371	1		Any A90/A91, complete years 2017-2023	2017-2023	196	82	15,876	837	93.225	0.142	1	1
M6-1	7,998	86	687	687	1.287	1		Broad ICD-only syndrome using any group except sepsis	2017-2024	196	86	18,228	687	94.786	0.119	1	1
M6-2	7,533	81	584	584	1.295	1		Broad ICD-only syndrome using any group except respiratory failure	2017-2024	196	81	18,228	584	95.128	0.111	1	1
M6-3	8,091	87	728	728	1.304	1		Broad ICD-only syndrome using any group except renal failure	2017-2024	196	87	18,228	728	94.549	0.115	1	1
M6-4	8,091	87	727	727	1.318	1		Broad ICD-only syndrome using any group except hepatic failure	2017-2024	196	87	18,228	727	94.562	0.116	1	1

Supplementary Table S16. Information availability for home deaths and manual coding audit.

reviewer_question	variable_found	coverage_percent	usable	note
Laboratory confirmation	No	0	No	Not present in supplied SINADEF extract/dictionary
Previous clinical evaluation	No	0	No	Not present
Previous hospitalization	No	0	No	Not present
Clinical diagnosis route	No	0	No	Cause text is not a diagnosis-route variable
Certifying physician	No	0	No	Not present
Medical-record access	No	0	No	No linkage to clinical records
Autopsy field	Yes	100	Descriptive only	Does not validate dengue etiology
ICD-10 position and text	Yes	100	Yes	Supports certification-pattern comparison

Manual review summary

metric	n
sample_rows	146
unique_certificates	146
final_reviews	146
pending_reviews	0
false_positives	0
false_negatives	0
discrepancies	0
unadjudicated_discrepancies	0

Supplementary Table S17. Descriptive recorded deaths per 1,000 notified cases by department, 2017–2024.

Department	Deaths	Notified cases	Severe cases	Deaths per 1,000 cases
PIURA	222	172,768	658	1.285
LAMBAYEQUE	113	46,915	307	2.409
MADRE DE DIOS	103	19,762	202	5.212
ICA	99	68,294	179	1.45
SAN MARTIN	58	34,044	152	1.704
LORETO	58	41,888	201	1.385
JUNIN	52	20,649	74	2.518
LIMA	48	121,496	287	0.395
LA LIBERTAD	47	83,520	220	0.563
UCAYALI	41	27,986	170	1.465
TUMBES	35	28,145	75	1.244
AMAZONAS	18	11,971	24	1.504
ANCASH	18	34,366	84	0.524
CAJAMARCA	17	19,045	26	0.893
CUSCO	9	13,572	34	0.663
AYACUCHO	8	7,039	14	1.137
HUANUCO	8	8,749	28	0.914
PASCO	5	3,371	19	1.483
PUNO	3	629	0	4.769
HUANCAVELICA	3	0	0	
CALLAO	2	8,138	21	0.246
TACNA	1	0	0	
AREQUIPA	0	28	0	0
APURIMAC	0	11	0	0
MOQUEGUA	0	1	0	0

These are aggregated descriptive ratios and are not individually linked case-fatality estimates.

Supplementary Table S18. Reproducibility and quality-assurance summary.

QA domain	Passed/observed	Expected/total	Interpretation
Canonical validation controls	83	83	All passed in approved package
Negative validator mutations	11	11	All returned nonzero and the nominal control failed
Deterministic artifacts identical	90	90	Two clean runs
Analytical blocks/fronts approved	9	9	Final acceptance checklist
Certificate manual reviews completed	146	146	No pending or unadjudicated discrepancies

Supplementary Table S19. Full-year 2024 reconciliation across analytical populations and surveillance.

month	certificates_any	territorial_eligible	panel_deaths	model_deaths	surveillance_cases	surveillance_severe_cases
1	7	6	6	6	12,138	52
2	31	31	31	31	37,142	116
3	56	56	56	56	69,337	176
4	14	14	14	14	96,233	243
5	9	9	9	9	32,247	76
6	2	2	2	2	7,495	19
7	0	0	0	0	2,939	14
8	0	0	0	0	1,699	5
9	1	1	1	1	2,164	7
10	2	2	2	2	2,178	9
11	1	1	1	1	3,901	7
12	0	0	0	0	4,058	12

Supplementary Figure S1. Corrected sequential study flow for the updated analysis.

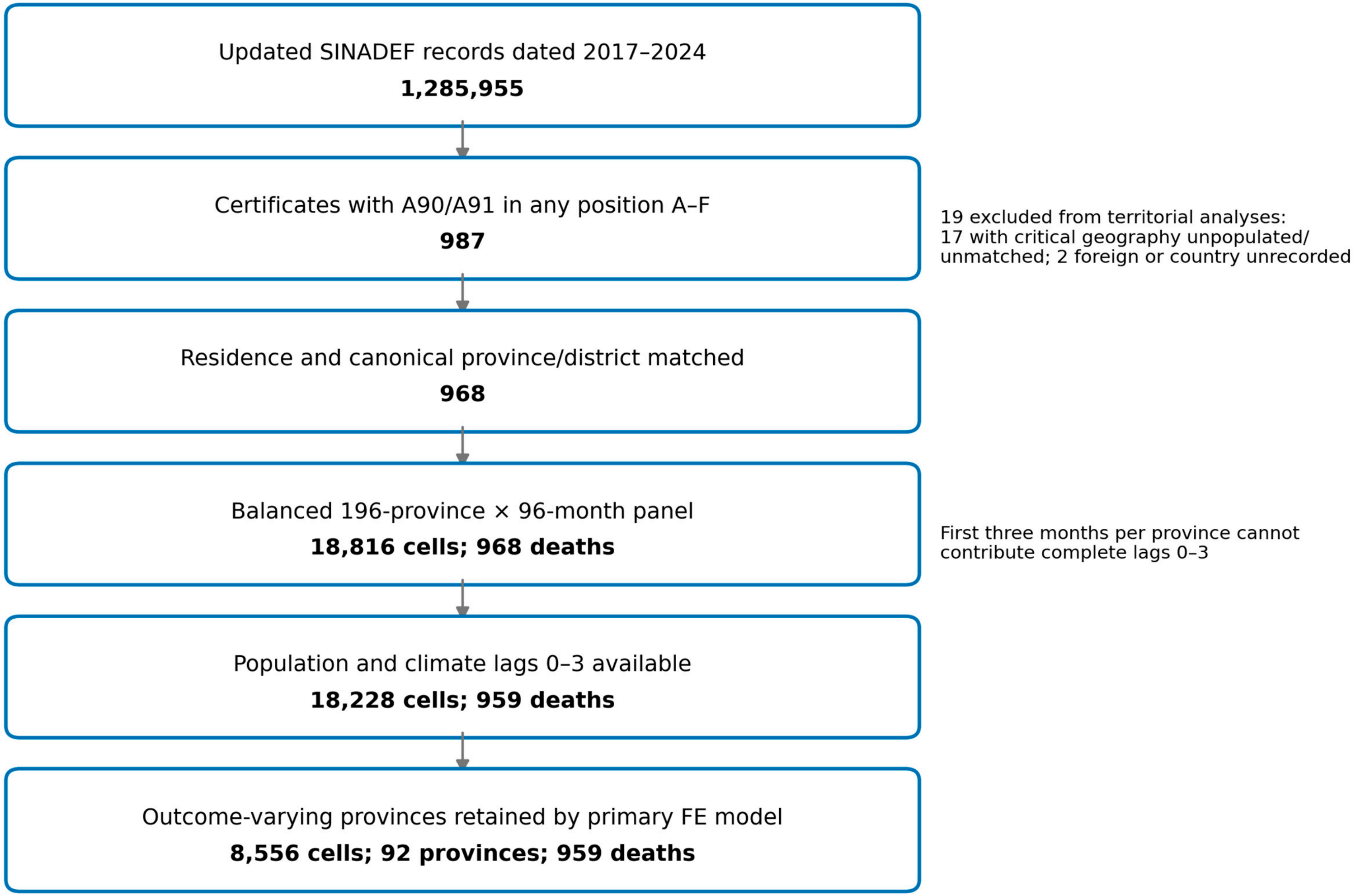

The 19 geographic exclusions and the three lag-construction months per province are represented separately.

Supplementary Figure S2. Conceptual directed acyclic graph for interpretation of climate, transmission, care, death, certification, and surveillance.

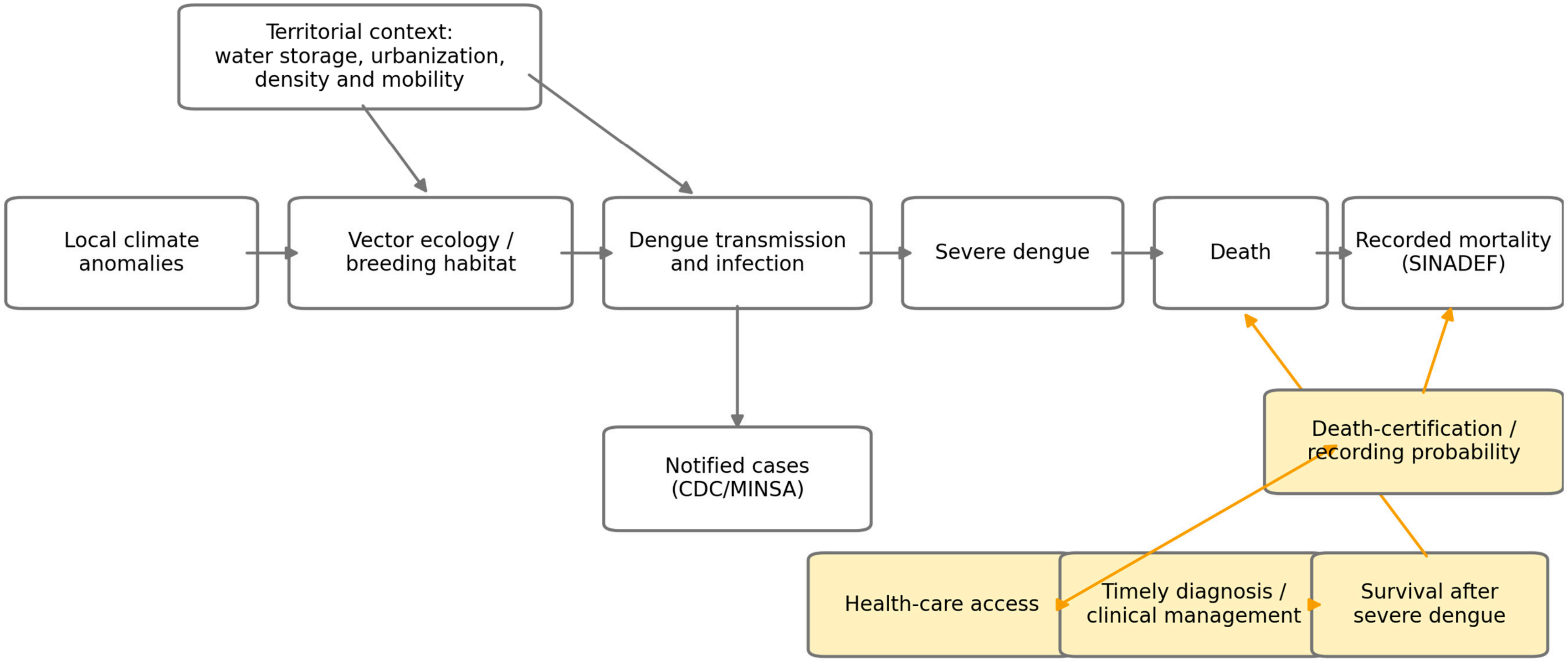

The diagram guides interpretation and does not establish individual causal effects.

Supplementary Figure S3. ICD-10 cause-position population and coding depth by year.

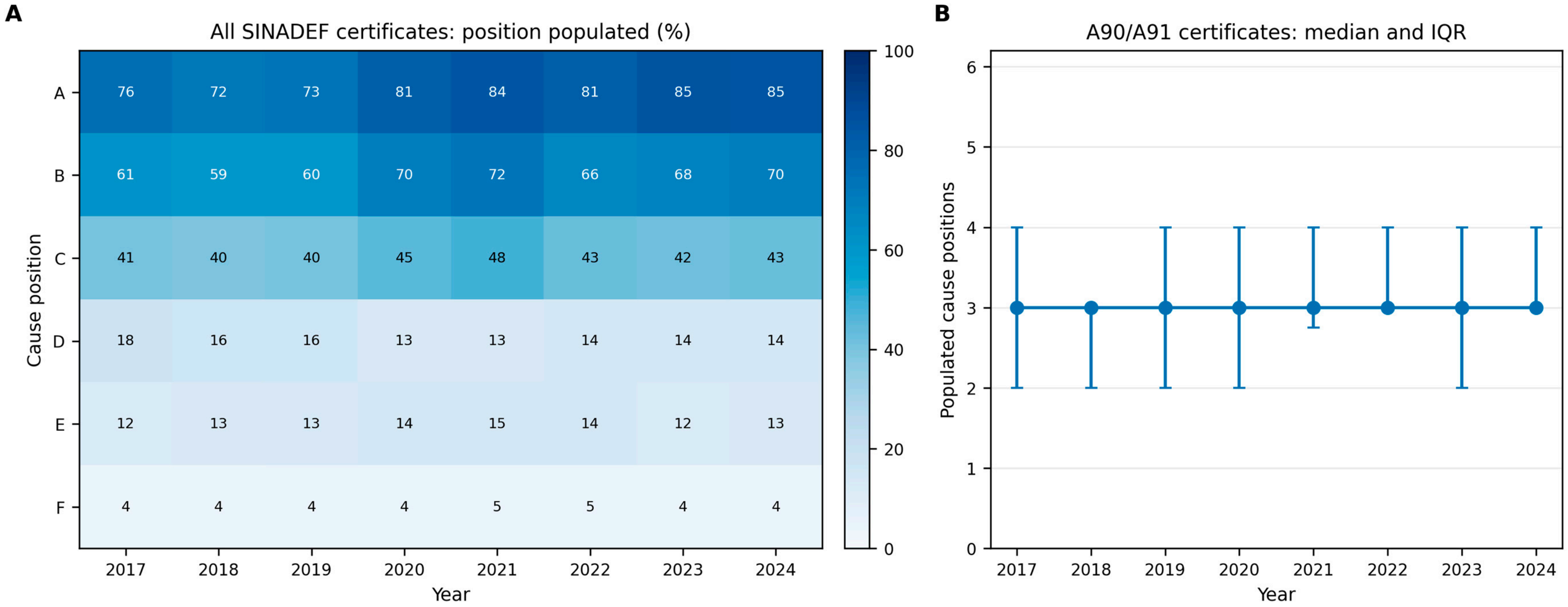

Unpopulated later positions are not automatically treated as missing data.

Supplementary Figure S4. Construction of the corrected province-month analytical panel.

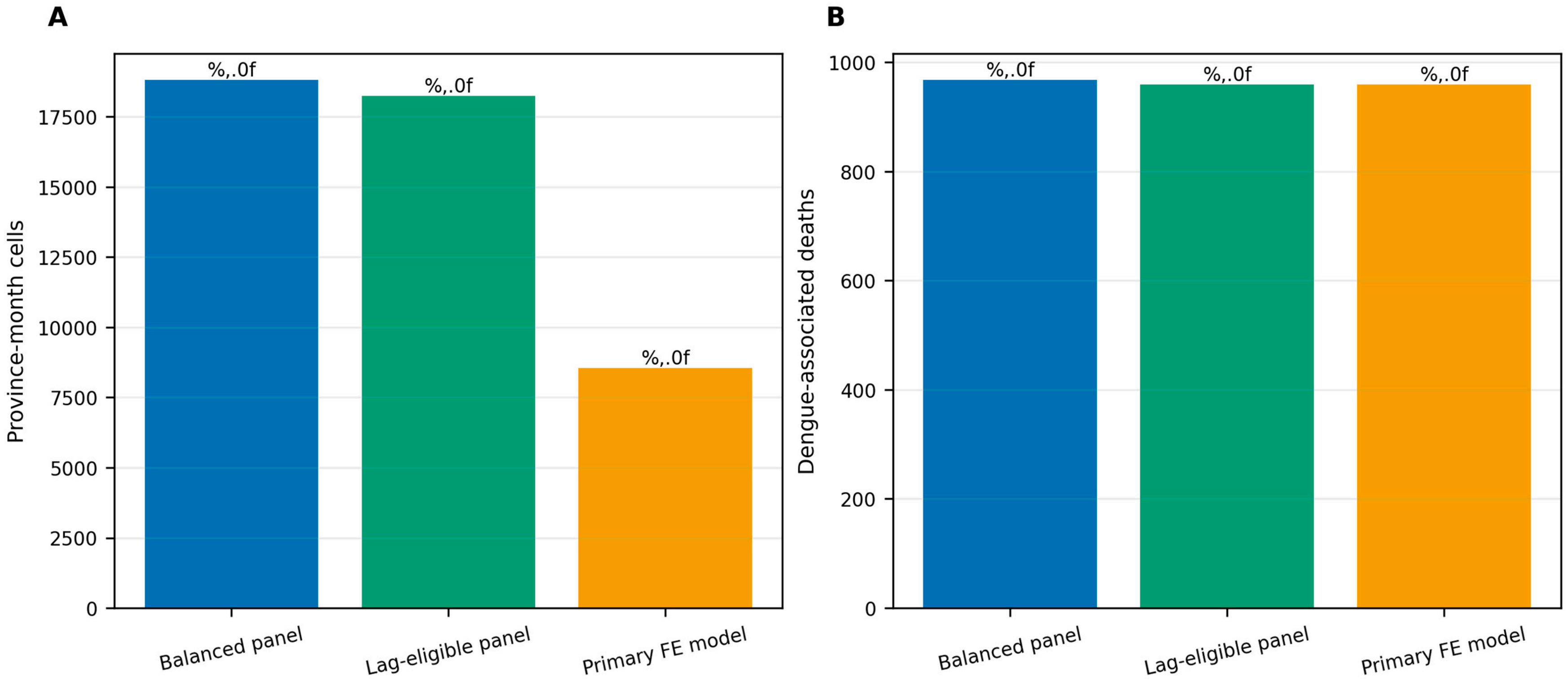

Supplementary Figure S5. Correlations among standardized climate anomalies in the corrected panel.

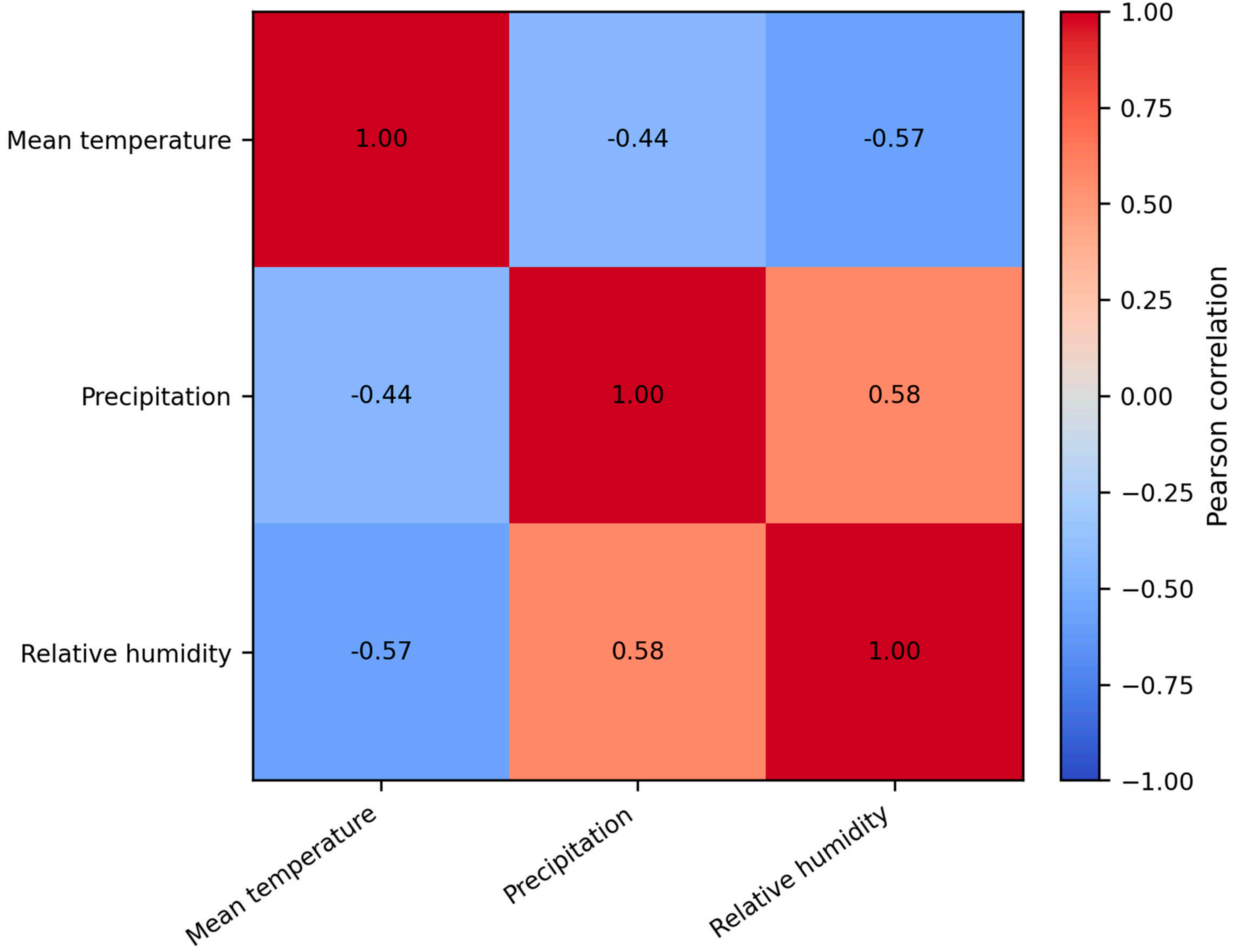

Supplementary Figure S6. Monthly outcome definitions over the complete 96-month calendar skeleton.

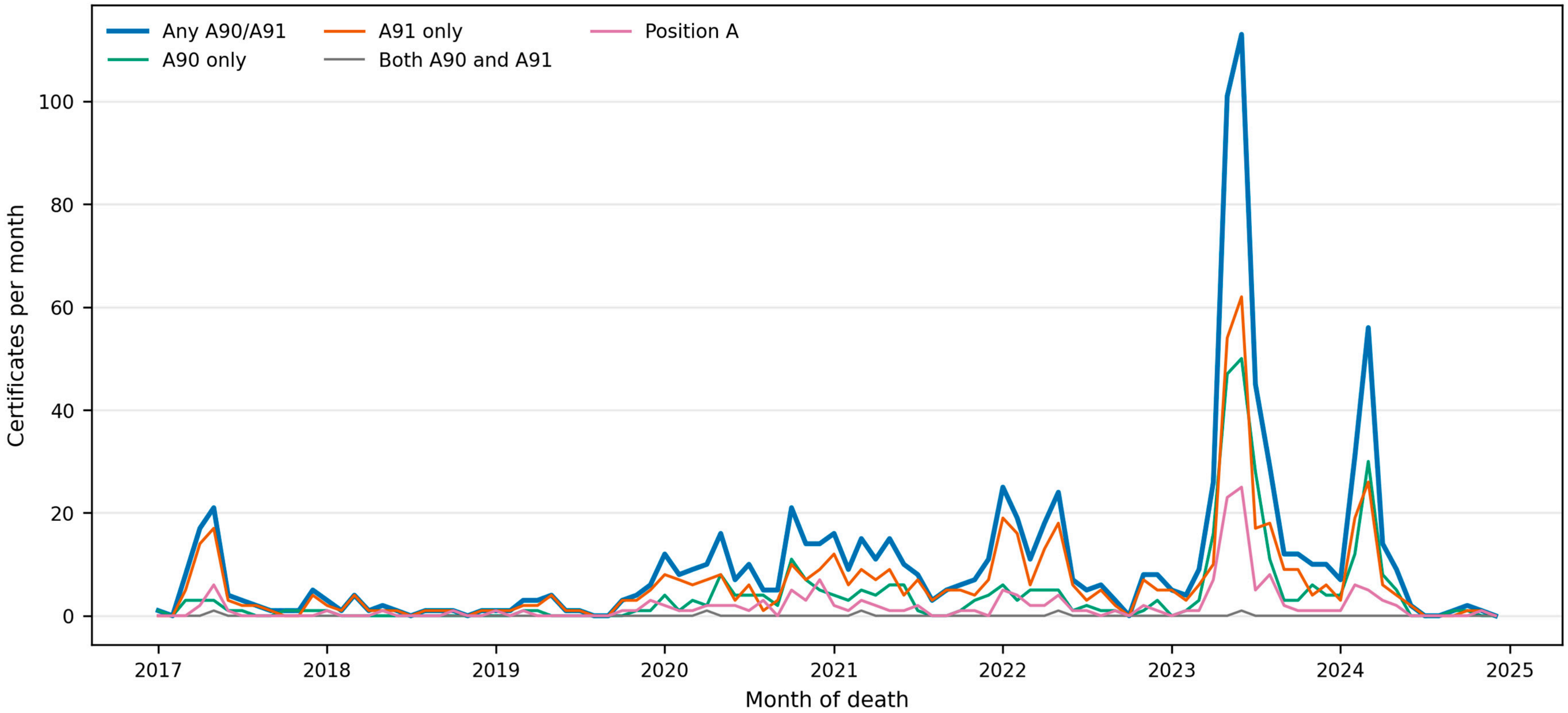

All zero-event months are explicitly represented.

Supplementary Figure S7. Lagged national concordance between notified cases and dengue-associated deaths.

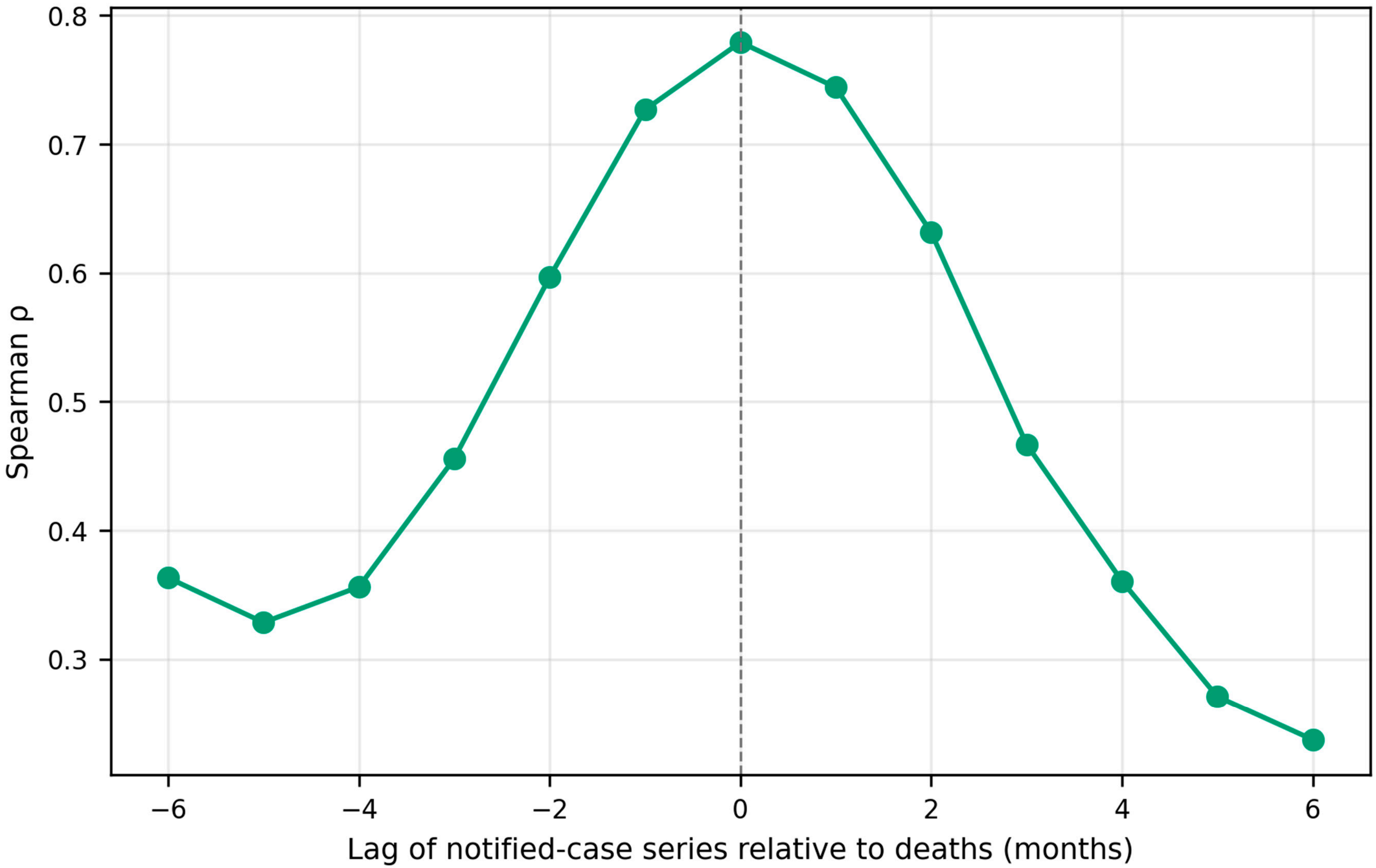

Positive lag values indicate the notified-case series shifted relative to deaths as labeled on the axis.

Supplementary Figure S8. Leave-one-component-out sensitivity of the broad ICD-only syndromic outcome.

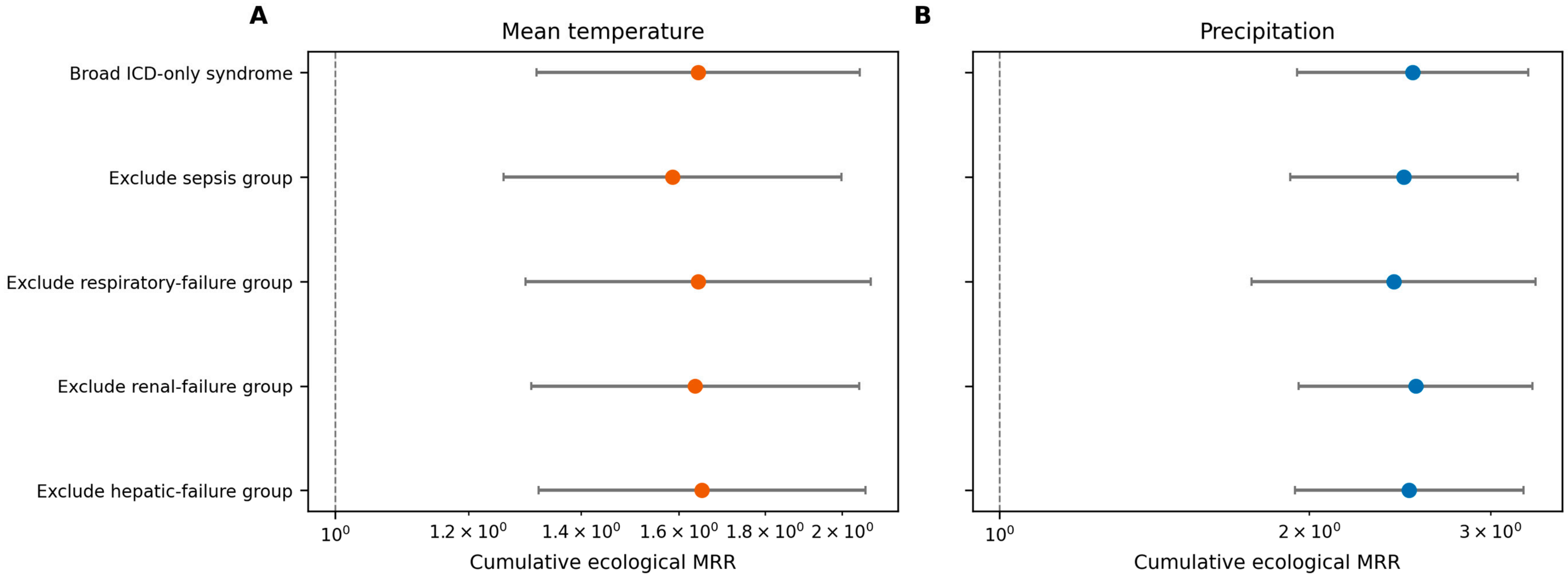

Supplementary Figure S9. Diagnostics across the corrected primary and sensitivity models.

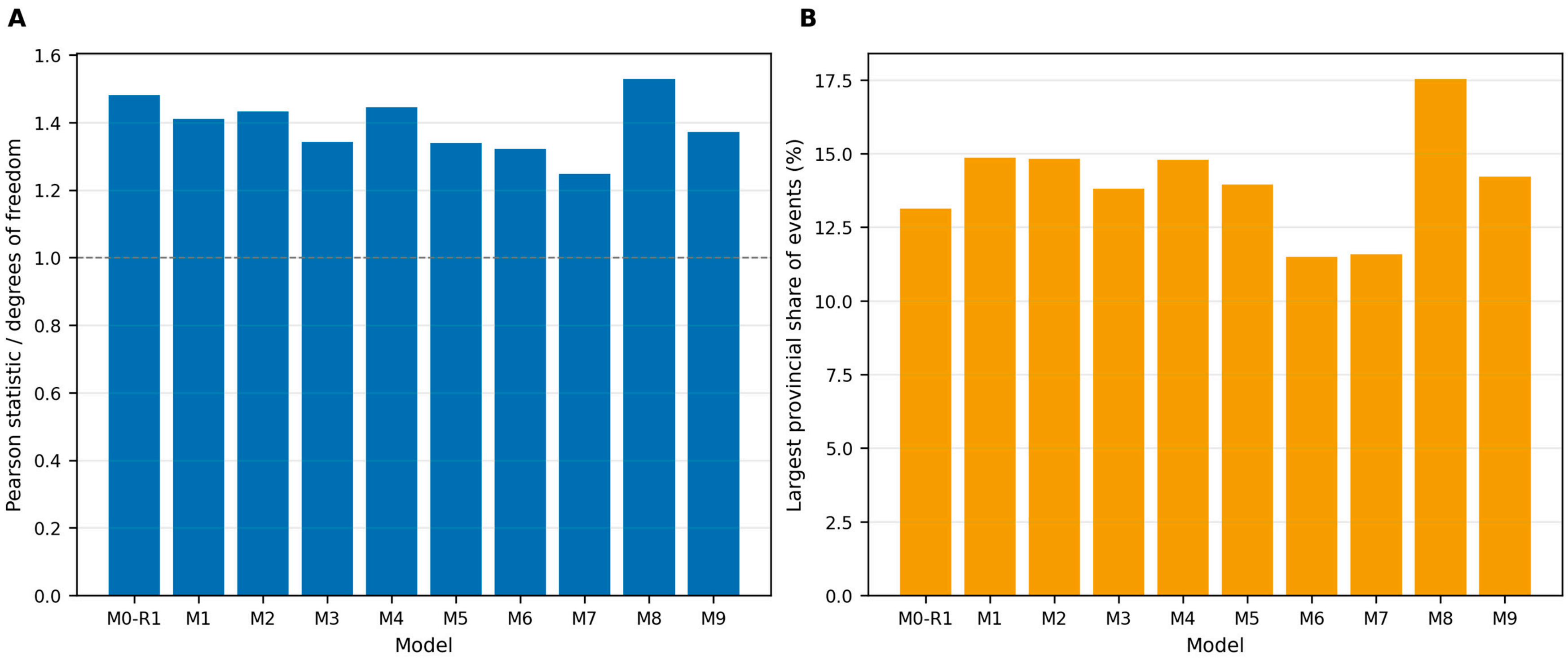

Supplementary Figure S10. Sensitivity of the fixed-province geographic classification.

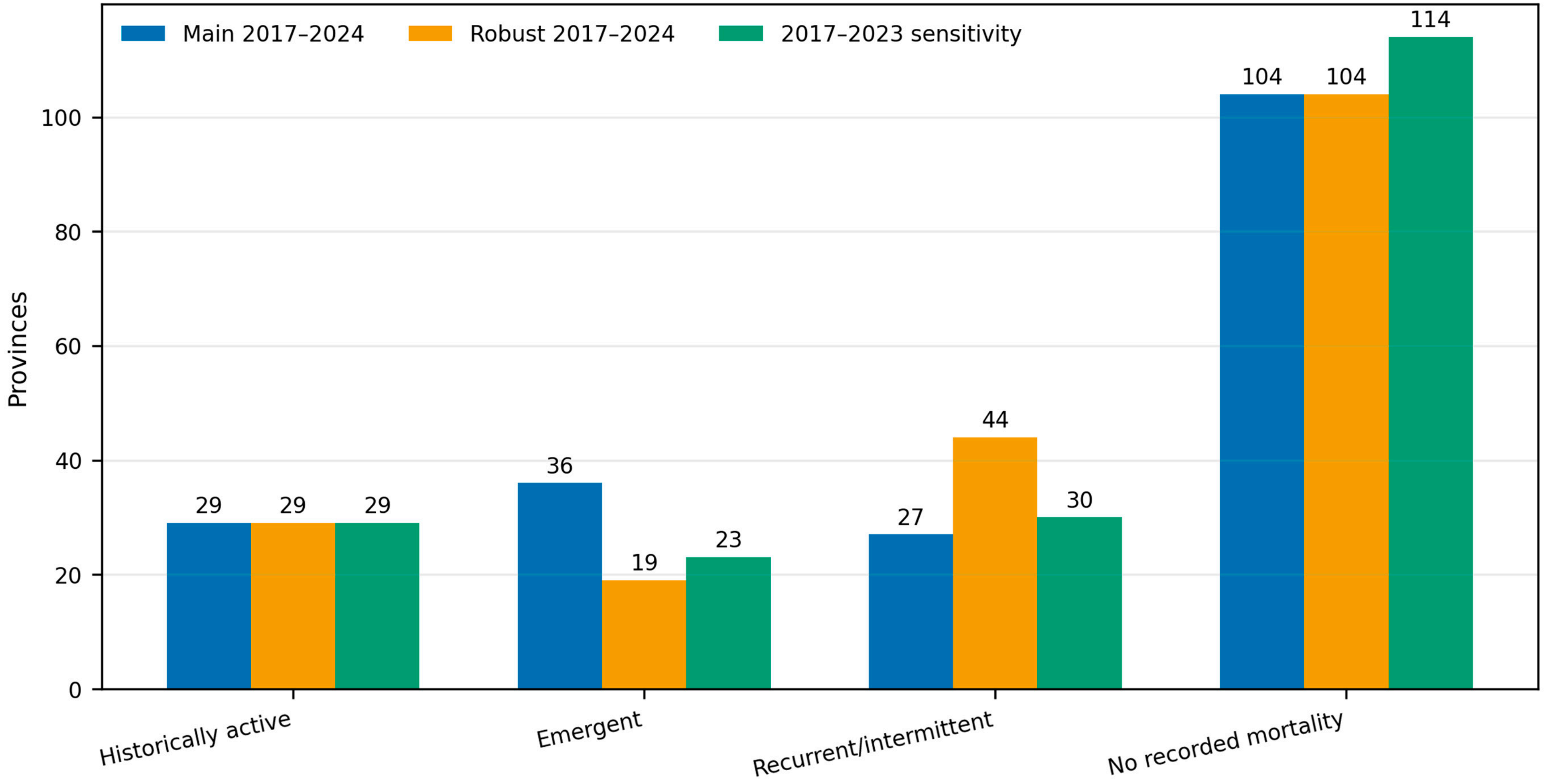

Supplementary Figure S11. Full-year 2024 outcome reconciliation by month.

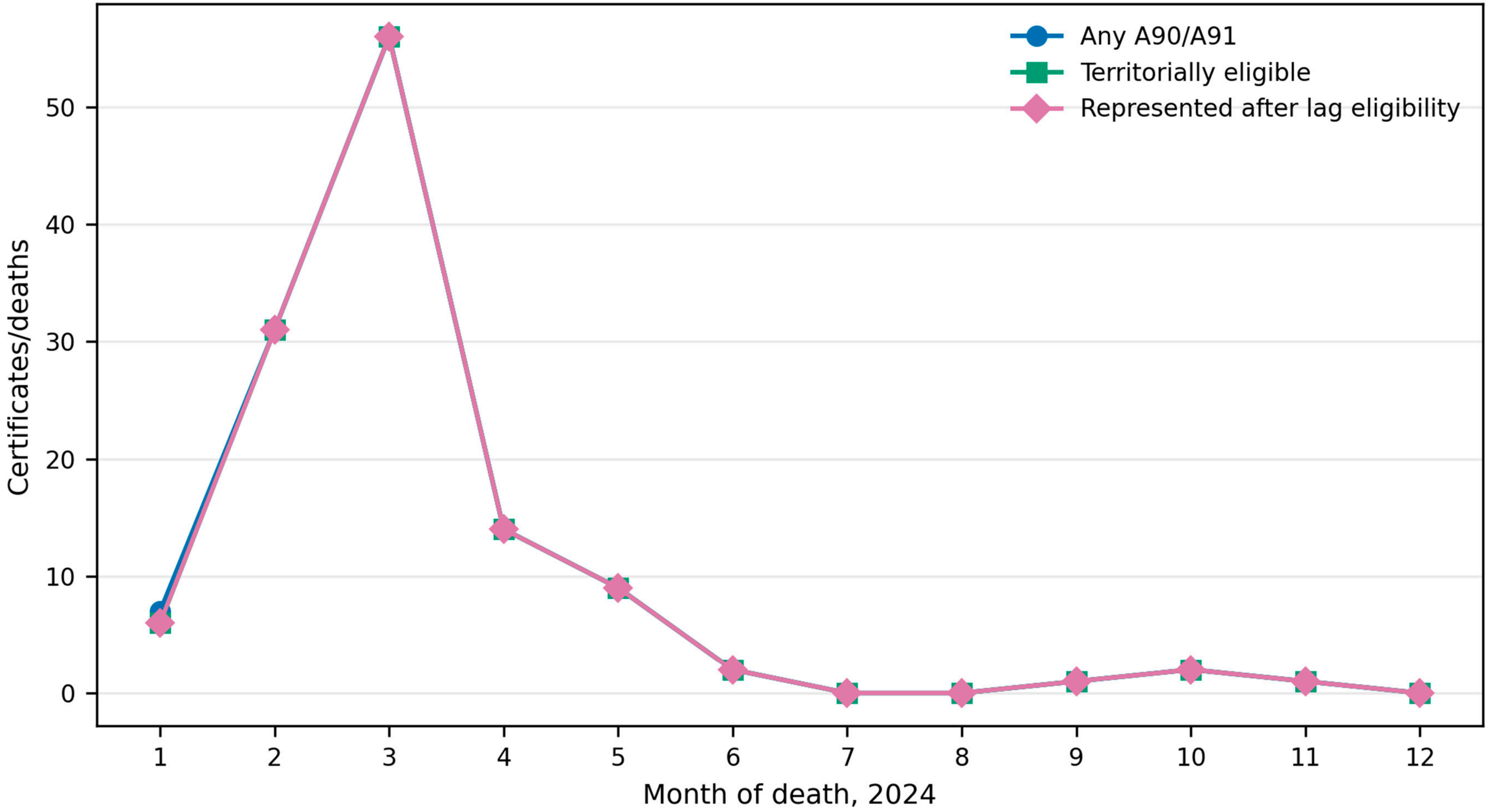

The updated snapshot contains records for all 12 calendar months; administrative exhaustiveness cannot be certified.

Supplementary Figure S12. Available certification characteristics for home and health-facility deaths.

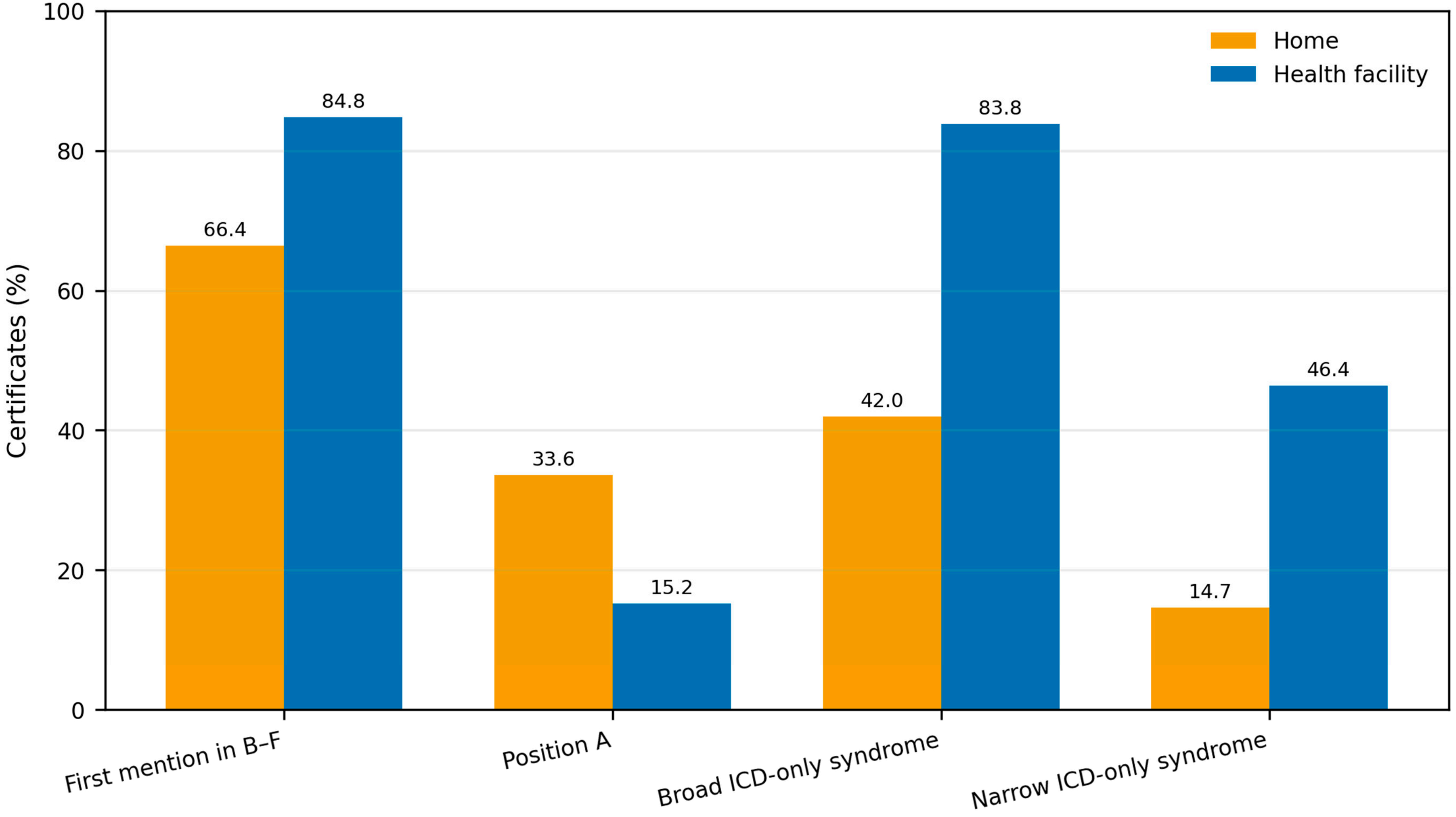

Clinical confirmation and prior-care variables were not available.
